# Insertion Specificity of the hATx-6 Transposase of *Hydra magnipapillata*

**DOI:** 10.3389/fmolb.2021.734154

**Published:** 2021-12-20

**Authors:** Paul Riggs, George Blundell-Hunter, Joanna Hagelberger, Guoping Ren, Laurence Ettwiller, Mehmet Berkmen

**Affiliations:** New England Biolabs, Ipswich, MA, United States

**Keywords:** DNA cut and paste transposons, transposable element, target site duplication, hAT transposases, HERMES, assessing target site specificity

## Abstract

Transposable elements (TE) are mobile genetic elements, present in all domains of life. They commonly encode a single transposase enzyme, that performs the excision and reintegration reactions, and these enzymes have been used in mutagenesis and creation of next-generation sequencing libraries. All transposases have some bias in the DNA sequence they bind to when reintegrating the TE DNA. We sought to identify a transposase that showed minimal sequence bias and could be produced recombinantly, using information from the literature and a novel bioinformatic analysis, resulting in the selection of the hATx-6 transposase from *Hydra vulgaris* (aka *Hydra magnipapillata*) for further study. This transposase was tested and shown to be active both *in vitro* and *in vivo,* and we were able to demonstrate very low sequence bias in its integration preference. This transposase could be an excellent candidate for use in biotechnology, such as the creation of next-generation sequencing libraries.

## Introduction

Transposable elements (TEs) are defined as “DNA sequences that are able to move from one location to another in the genome (Munoz-Lopez and Garcia-Perez, 2010) and are commonly found in genomes of almost every known organism” (Aziz et al., 2010). TE’s can be divided into two groups, retrotransposons and DNA transposons. Retrotransposons mobilize with an RNA intermediate step and generally have more complex mechanisms, while DNA TE’s do not require RNA intermediates, and are often simpler systems. In many cases DNA transposition is catalyzed by a single protein, which performs the entire excision and reintegration steps; these enzymes are known as a transposases.

This capacity to perform all the reaction steps makes the transposases useful tools for genomic editing applications. One of the earliest applications was random mutagenesis, where TE’s would be inserted into the genome at random sites to discover phenotypes associated with the insertion mutations (Goryshin et al., 2000). TE’s can also be used to insert genes in a targeted manner, into the genomes of various organisms. The most common examples, particularly in vertebrates, are *piggyBac* and *Sleeping Beauty* (Gao and Liu, 2017; Qing et al., 2017). Recently, TE’s in the Tn7 family that contain Cas subtype I-F and subtype V-K targeting modules, as well as through fusions of Cas-9 to the Hsmar1 transposase, have been used to mediate targeted gene insertions in *E. coli* (Bhatt and Chalmers, 2019; Klompe et al., 2019; Strecker et al., 2019).

**FIGURE 1:**
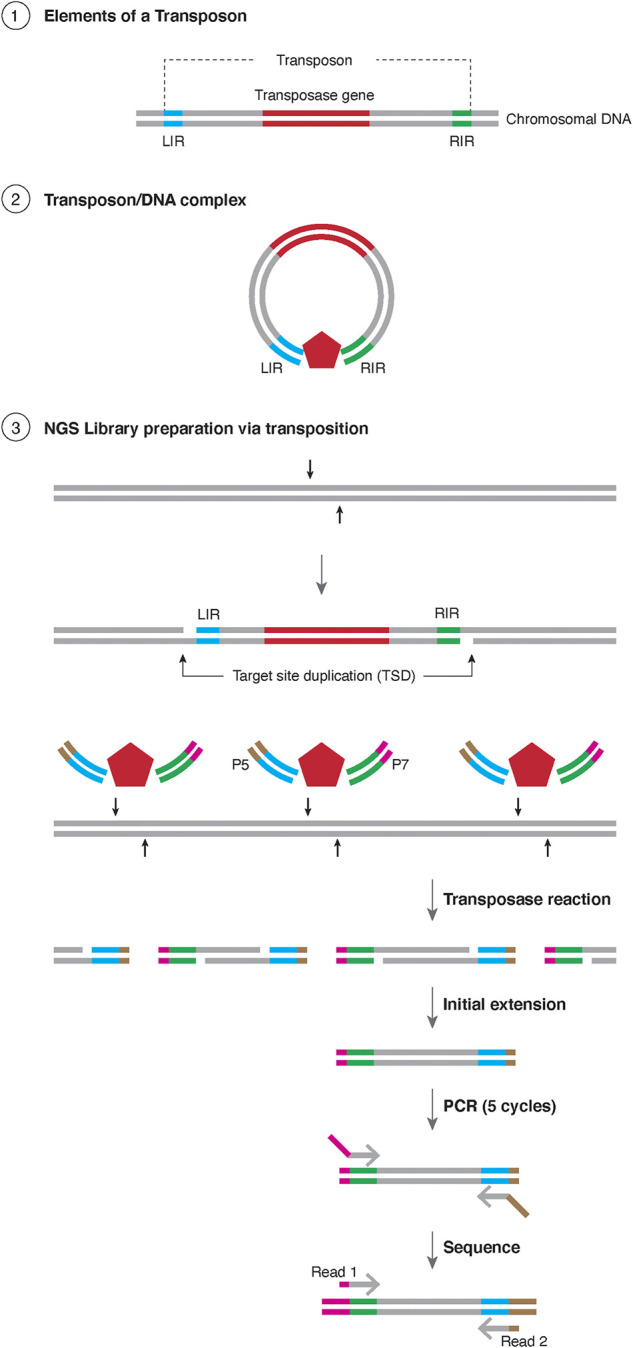
Schematic representation of Tagmentation ① Representation of the elements of a transposon, with the transposase gene in red and the sequences for the Left Inverted Repeat (LIR) in blue and right inverted repeat (RIR) in green. The chromosome is represented as black lines. ② insertion of a transposon element resulting in Target Site Duplication (TSD). ③ Schematic representation of Tagmentation. A transposase (red pentagon) is in complex with DNA containing LIR and RIR, each containing either the sequence for Illumina P5 (brown) or P7 (magenta) sequences. The resulting transposase reaction results in TSD which is filled with initial extension, amplified for 5 rounds with PCR and the products are sequenced bidirectionally with Illumina primers.

The most common application for transposases currently is in the construction of libraries for next-generation sequencing (NGS) (Adey et al., 2010). To generate a library, the genomic DNA of the sample needs to be fragmented into small oligonucleotides followed by ligation of specific adapters to the cleaved ends in order to amplify and sequence the library. Classically, the cleavage of the genomic DNA was achieved by physical fragmentation using sonication or enzymatically by endonuclease digestion, while the adapter ligation required a separate enzymatic reaction. Alternatively, one can use a transposase to perform the cleavage of the genomic DNA and the adapter ligation can be conducted in a single reaction (Figure 1). To construct an NGS library, first oligonucleotides (containing TE specific end sequences and an adapter) are mixed with the transposase (both Tn5 and MuA based systems are used commercially, as Nextera^™^ and MuSeek^™^, respectively), resulting in a transposase-DNA complex. These complexes are mixed with the purified genomic DNA, where the transposase integrates the transposon ends (with adapters attached) into the genomic DNA. As the two oligonucleotides are not connected to each other, a break is created in the genomic DNA.

The most widely used transposase for multiple applications (random mutagenesis, ATAC-seq, NGS library prep (Buenrostro et al., 2015; Gao and Liu, 2017)) is Tn5, first isolated from the genome of *Escherichia coli* (Berg et al., 1975). Tn5 transposase is currently the most studied system and was the first transposase to have its crystal structure solved (Davies et al., 2000). The version of the transposase used in applications has been modified from the wild-type form with several mutations (Wiegand and Reznikoff, 1992; Weinreich et al., 1994; Zhou and Reznikoff, 1997; Goryshin and Reznikoff, 1998). The E54K mutation improves the binding of the transposase to the TE ends. M56A does not directly affect activity but does prevent the expression of an N-terminally truncated form of the transposase (the inhibitor protein) which can bind and inhibit the functional transposase. The final mutation L372P increases activity by reducing the flexibility of the C-terminal portion of the transposase, which in the wild-type form interferes with the N-terminal portion as it binds to the TE end sequence. This hyperactive Tn5 shows a GC bias in insertion site preference, though a more recent study has used a series of four further mutations to partially reduce the bias (Green et al., 2012; Kia et al., 2017). The bias of Tn5 does not inhibit its use with genomes of GC content 30% or higher but is a significant limitation below that threshold (Kia et al., 2017).

In this study we sought to identify and characterize of a new transposase in a novel approach utilizing bioinformatic tools. We took the data set from Arensburger 2011 as a starting point, which collated 299 TE’s of the hAT (*hobo/Ac/Tam3*) superfamily (Arensburger et al., 2011), which was first discovered in plants (Kempken and Windhofer, 2001). hAT transposases are attractive targets as some members of this superfamily have been demonstrated to have low target site specificity (Vollbrecht et al., 2010; Lazarow et al., 2012; Michel et al., 2017). Our goal was to identify an unknown transposase with low target site preference from this dataset, that could be purified, characterized, and used in NGS. Analyzing the predicted insertion sites bioinformatically, several candidate transposases were identified. After selecting the 6th putative transposase (hATx-6), which had the lowest predicted site specificity, we purified the candidate transposase by over expressing it in *E. coli*. We characterized the transposase through *in vivo* papillation assays in *E. coli* and *in vitro* transposition assays. We then analyzed the insertion site preference using genomic DNA with various GC contents, to determine the insertion site preference, and its viability for NGS library preparation.

## Materials and Methods

### Materials

We grew cells in Rich Media (10 g/L Tryptone, 5 g/L Yeast Extract, 5 g/L NaCl, NaOH to pH 7.2) or in LB (10 g/L Tryptone, 5 g/L Yeast Extract, 10 g/L NaCl, 10 mM MgCl2 NaOH to pH 7.2). Restriction enzymes, HiFi master mix, and NEBNext Ultra II FS kit were all supplied by New England Biolabs (NEB). *E. coli* and *Rhodopseudomonas palustris* genomic DNA was prepared by Lofstrand Labs Limited and human NA19240 was purchased from the Coriell Institute (Llamas et al., 2019). *Haemophilus influenzae* genomic DNA was the kind gift of Keith Lunnen (NEB).

### Bioinformatics

**FIGURE 2:**
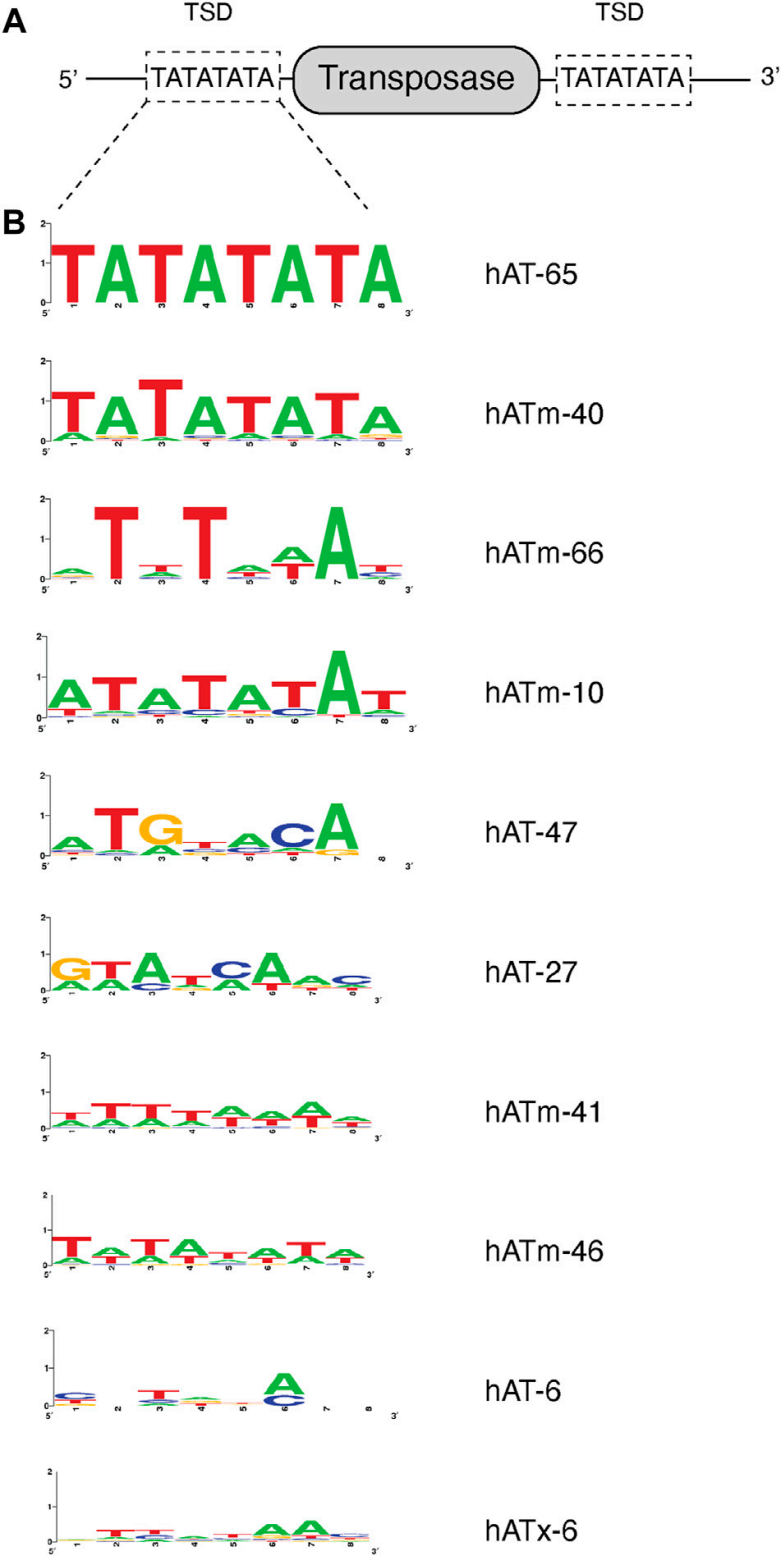
Predicted target site preference of transposable elements. **(A)** Schematic representation of the 8 nt target site duplication (TSD). Flanking DNA sequences of predicted transposases within the genome are analyzed and the TSD at 5′ and 3′ of the transposase are identified. The TATATATA TSD of hATm-3 is shown as an example. **(B)** Predicted 8 nt TSD of various transposases in order of TSD sequence bias shown as weblogo.

Biopython scripts used are shown in Supplementary Table S1. Previously published data on the target site duplication indicated that a number of TE’s in the genomes of cnidarians had low target site specificity (Arensburger et al., 2011). We constructed scripts that searched for hAT TE’s in the genome of *Hydra vulgaris* (BioProject PRJNA12876), using a TE end as the query for a BLAST search. We then examined the target site duplication (TSD) to see if at least seven of the eight bases were identical at each end of a putative insertion in the genome. If true, a weblogo of the sequence was generated. We selected TE’s that had weblogos indicating low site preference in the TSD for further study (Figure 2).

To identify the transposase gene, we used a modified script that extracted the sequences of the TE’s. We examined the sequences for open reading frames that 1) were sufficient size to encode a hAT transposase (∼500 amino acids), 2) had homology to a hAT transposase by BLAST, and/or 3) contained the “hAT dimerization domain” (pfam 05699) when submitted to HMMR (https://www.ebi.ac.uk/Tools/hmmer). We lined up the candidate sequences in SeaView (Gouy et al., 2010) and derived a consensus sequence for the transposase (Supplementary Figure S1).

We analyzed sequencing results by first identifying and caching reads that had the 20 base-pair left end sequence of the hATx-6 TE (see DNA sequencing below), and removing the 20 bases, with the program cutadapt (Martin, 2011). We then used a Python script to create a position weight matrix of the first 28 bases at the end of each sequence, the first 8 bases being the TSD and the following 20 bases comprising the flanking region.

### Bacteria and Plasmids

We used NEB 10-beta (NEB C3019) as a host for cloning and expression, and T7 Express for the papillation assay (NEB C2566).

We composed a DNA sequence that encoded the hATx-6 transposase suitable for expression in *E. coli* using the Codon Optimization tool from IDT (https://www.idtdna.com/CodonOpt). We then divided the gene sequence into three sections with 20 base overlaps and had the sections synthesized (IDT gBlocks) (Supplementary Figure S2). The gene was assembled with the vector using the NEBuilder HiFi assembly Master Mix (NEB E2621). We then cloned the transposase into pMAL-c5X cut with XmnI and SbfI, assembling with a PCR fragment made using primers pMAL-c5X-hatx-6-F and pMAL-c5X-hatx-6-R. We named the plasmid pMAL-c5X-hATx-6 (Supplementary Figure S2).

Oligonucleotides are listed in Supplementary Table S2. We constructed the TE donor for the papillation assay by first assembling a PCR fragment made using pSYX20 (Morgan et al., 1996) as a template and primers pSYX20 F1330 and pSYX20 R4827 with a PCR fragment of *lacZ* made with primers clacZ_fwd2 and clacZ_rev2, using the NEBuilder HiFi assembly master mix. We then added 30 base-pairs of the right and left ends of the hATx-6 TE to the resulting plasmid by Q5 site-directed mutagenesis in two steps, first with the primers hATx-6-RE30-pSYXlac fwd and hATx-6-RE30-pSYXlac rev for the right end, then the primers hATx-6-LE30-pSYXlac fwd and hATx-6-LE30-pSYXlac rev for the left end, flanking the *lacZ* gene (Supplementary Table S2).

### hATx-6 Transposase Expression

We first did small-scale expression trials by transforming NEB 10-beta with the expression plasmid and inoculating 10 ml LB containing 100 μg/ml ampicillin and incubating at 37°C overnight. We then inoculated 20 ml of the same medium with 0.2 ml of the overnight culture and grew to an OD600 = 0.5. We added IPTG to 0.3 mM, separated the culture into 4 × 5 ml aliquots and incubated them at 37°C for 2 h, 30°C for 4 h, 25°C for 8 h and 16°C for 16 h. In a separate experiment, we grew an overnight as described above and inoculated 5 ml with 0.05 ml of the overnight. We incubated the culture at 37°C until it reached an OD600 of 0.5, then shifted it to 18°C for 16 h. In both cases, we harvested the cells by centrifugation then lysed by detergent lysis as described (Walker et al., 2010). We analyzed samples by SDS-PAGE and looked for a band of the appropriate size.

### hATx-6 Transposase Purification

Overnights grown at 37°C on a roller drum were used to inoculate 60 ml rich media in a 250 ml flask. This inoculum was used to inoculate 6 L of Rich + ampicillin of NEB 10-beta [pMAL-c5X-hATx-6] and incubated at 37°C until the culture reached an OD600 of 0.5, then shifted the culture to 18°C and incubated overnight. We harvested the cells at 4,000 × g, resuspended them in 25 ml of 20 mM Tris-HCl pH 7.5, 0.25 M NaCl, 0.2 mM TCEP, 0.1 mM EDTA and froze them at −20°C. The following day, we sonicated them for 3 × 2 min on a 50% duty cycle with a Heat Systems sonicator, for a total of 3 min sonication time. We pelleted cell debris at 20,000 × g, then applied the supernatant to two 5 ml Heparin HiTrap columns connected in series (GE Life Sciences # 17040701). We eluted the column with a NaCl gradient from 0.25 to 1.0 M in the resuspension buffer. We identified fractions containing the MBP-hATx-6 protein by SDS-PAGE and pooled the fractions and applied them to a column containing 40 ml of Amylose Resin High Flow (NEB #8022). We washed the column with 120 ml of 20 mM Tris-HCl, 1 M NaCl, 0.2 mM TCEP, 0.1 mM EDTA, then eluted with the same buffer +10 mM maltose. We pooled the peak, concentrated it in a Vivaspin 20 spin concentrator with a 30 K MW cutoff (Sartorius VS2021) to ∼5 ml, and applied it to a HiPrep 26/60 Sephacryl S-300 HR column (GE Life sciences 17119601). The MBP-hATx-6 fusion protein and two truncated derivatives eluted as two poorly separated peaks directly after the void volume. We pooled the peaks, concentrated them to 5 ml in a Vivaspin 20 concentrator, then dialyzed the sample overnight vs. 20 mM Tris, 0.5 M NaCl, 0.2 mM TCEP, 0.1 mM EDTA, 50% glycerol, pH 7.5, and stored at −20°C (Supplementary Figure S3).

### Transposase Activity Assay

We mixed 1–5 µl of MBP-hATx-6 at 9.2 μg/μl, 1.0 µM of TE end(s) and 50 ng of supercoiled pBR322 DNA in a 20 µl reaction containing 25 mM MOPS pH 7.0, 0.2 mM TCEP, 5 mM MgCl_2_, 50 μg/ml BSA, 100 mM NaCl, 0.1 mM EDTA and incubated at 30°C for 30 min. We stopped the reaction with 5 µl of 6x Purple Gel Loading Dye (NEB) and loaded 15 µl on a 1% agarose gel containing 0.5 μg/ml ethidium bromide. We measured activity by conversion of the supercoiled plasmid to linear and nicked forms. For assays where the TE ends were labelled with biotin, we followed transfer of the end to the plasmid DNA by electroblotting the DNA in the gel to a positively charged nylon membrane (Sigma # 11417240001) using a Bio-Rad Trans-Blot apparatus at 200 mA for 30 min. We washed and labelled the membrane using the Chemiluminescent Nucleic Acid Detection kit (ThermoFisher #89880) and visualized on a Amersham Typhoon Gel Imaging scanner (GE Life Sciences) with 488 nm laser and the Cy2 filter.

### Papillation Assay

We made competent cells from T7 Express containing the pSYX-RE30-lacZ-LE30 TE donor plasmid, then transformed with 1 ng of pMAL-c5X-hATx-6 transposase plasmid and plated on LB plates containing 0.1% lactose, 20 μg/ml X-Gal, 100 μg/ml ampicillin, diluted appropriately to obtain ∼100 colonies per plate. We incubated the plates at 37°C for 5 days, then stored them in the dark at 4°C overnight to enhance the X-Gal signal.

### DNA Sequencing

We first fragmented and tagged genomic DNA from *E. coli*, *Haemophilus influenzae*, *Rhodopseudomonas palustris* and human leukocytes in a tagmentation reaction containing 37 µg MBP-hATx-6, 50 ng genomic DNA, 0.1 µM LE20 in a 30 µl reaction containing 25 mM MOPS pH 7.0, 0.2 mM TCEP, 5 mM MgCl_2_, 50 μg/ml BSA, 100 mM NaCl, 0.1 mM EDTA. We purified the reaction using a Monarch DNA Clean-up kit and eluted the DNA in 30 µl of 10 mM Tris-Cl 0.1 mM EDTA pH 7.5. We then took 26 µl of this sample and took it through the NEBNext Ultra II FS workflow. The libraries were quantified using the NEBNext Library Quant kit for Illumina and sequenced on a MiSeq (Illumina).

## Results

### Screen for Transposases

**FIGURE 3:**
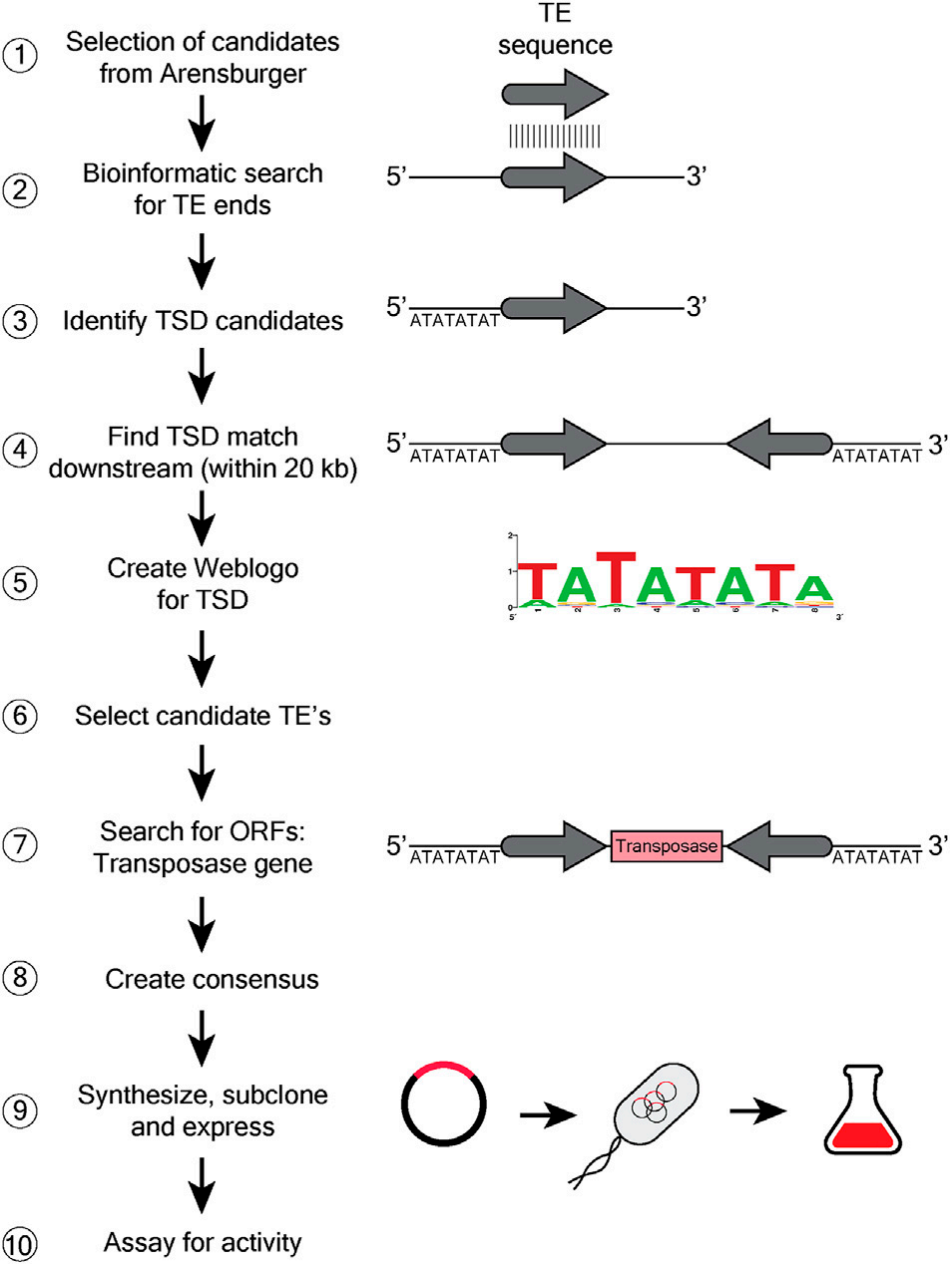
Bioinformatic search for discovery of new transposases. A schematic 10 step flow chart describing the steps followed to capture candidate transposases with low consensus TSD. The diagrams show general DNA as a black line, with the grey arrow indicating the transposon end (TE) sequence. The 8 letter sequence indicates the target site duplication (TSD). The transposase ORF is shown as a box with transposase written in it. ① Data set from Arensburger with 299 currated TE’s of the hAT (hobo/Ac/Tam3) superfamily (Arensburger et al., 2011) was used to search for unique transposases. ② TE sequence is searched throughout the genomic DNA, to identify matching sequences. ③ Identification of TSD sequences that would be directly adjacent to the TE sequence. ④ Identification of the other TE and TSD sequences downstream of the original. ⑤ Create a weblogo of TSD ⑥ and utilize the info to select for the appropriate TE ⑦ Identification of the transposase ORF within the transposon sequence. ⑧ A consenus sequence from homologs of the selected TE is created, ⑨ and the engineered synthetic TE is subcloned into an expression plasmid, transformed into *E. coli* and purified. ⑩ The purified TE is assayed for activity *in vitro*.

**FIGURE 4:**
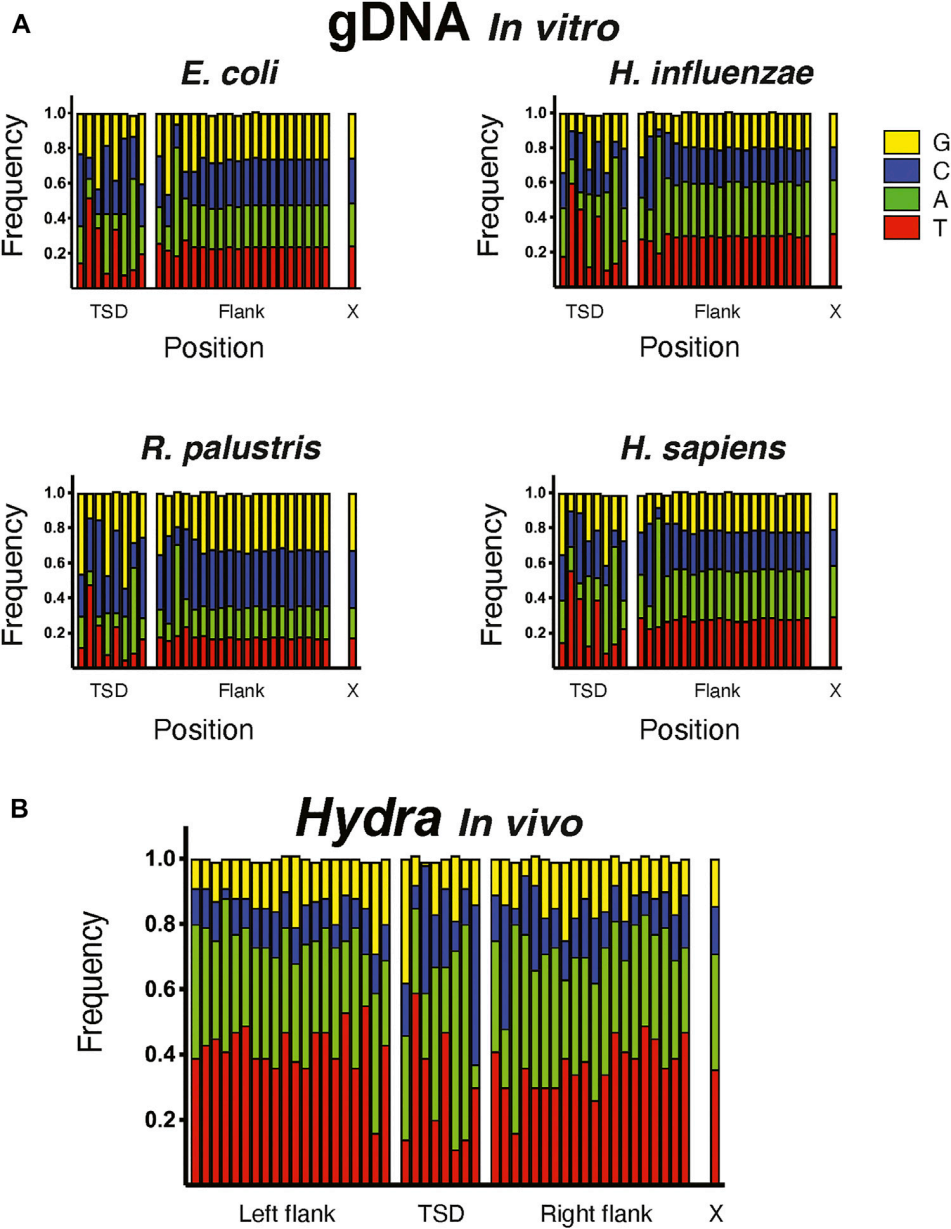
Target site preference of hATx-6 transposase. Graphs depicting the 8 bp target site duplication region (TSD), the 20 bp flanking the duplication site and X denotes the natural composition of nucleotides in the genome of the organism. G bases are shown in yellow, C in blue, A in green and T in red. **(A)** The insertion site preference of hATx-6 in *E. coli*, *H. influenzae*, *R. palustris*, and *H. sapiens* DNA. **(B)** The combined results of the sites found in the bioinformatic analysis of the Hydra genomic DNA.

We undertook a search for a hAT transposase with low specificity in target site selection, using a bioinformatic approach to assess the bias at the target site duplication created when the TE inserts at a new site (see Materials and Methods, Bioinformatics, Figure 3). This search led us to the hATx-6 TE in *Hydra vulgaris* (aka *Hydra magnipapillata*). A weblogo was constructed from the 54 insertions in the *Hydra* genome that had a recognizable target site duplication (TSD) using the genome composition as the base line (Figure 4B). This indicated low information content in all 8 bases of the TSD. Of these 54 hATx-6 elements in the genome, 10 had all or a portion of a 674 amino acid open reading frame that had an N-terminal Zn-finger BED domain (pfam 2892.15) and a C-terminal hAT dimerization domain (pfam 5699.14), indicating that it was the hATx-6 transposase (Arensburger et al., 2011). All of these open reading frames (ORFs) had multiple base differences that would create missense and frameshift mutations, making it unlikely that any of them could yield an active transposase (Supplementary Figure S1).

### Purification of hATx-6 Transposase

We created a consensus sequence (Supplementary Figure S1) from the 10 hATx-6 ORFs and assembled a gene encoding the putative consensus sequence transposase from synthesized oligonucleotides. We cloned the gene into pMAL-c5X and discovered that expression at 18°C overnight without induction resulted in the best yield of full-length MBP-transposase fusion protein. The protein prepared under these conditions was typically a mixture of full length fusion protein and one or two smaller truncation products, presumably C-terminal truncations as one step in the purification was binding of the N-terminal MBP to an amylose column. We obtained approximately 10 mg of MBP-hATx-6 from 6 L of culture, approximately half of which was full-length.

### *In vivo* and *In vitro* Activity of hATx-6

**FIGURE 5:**
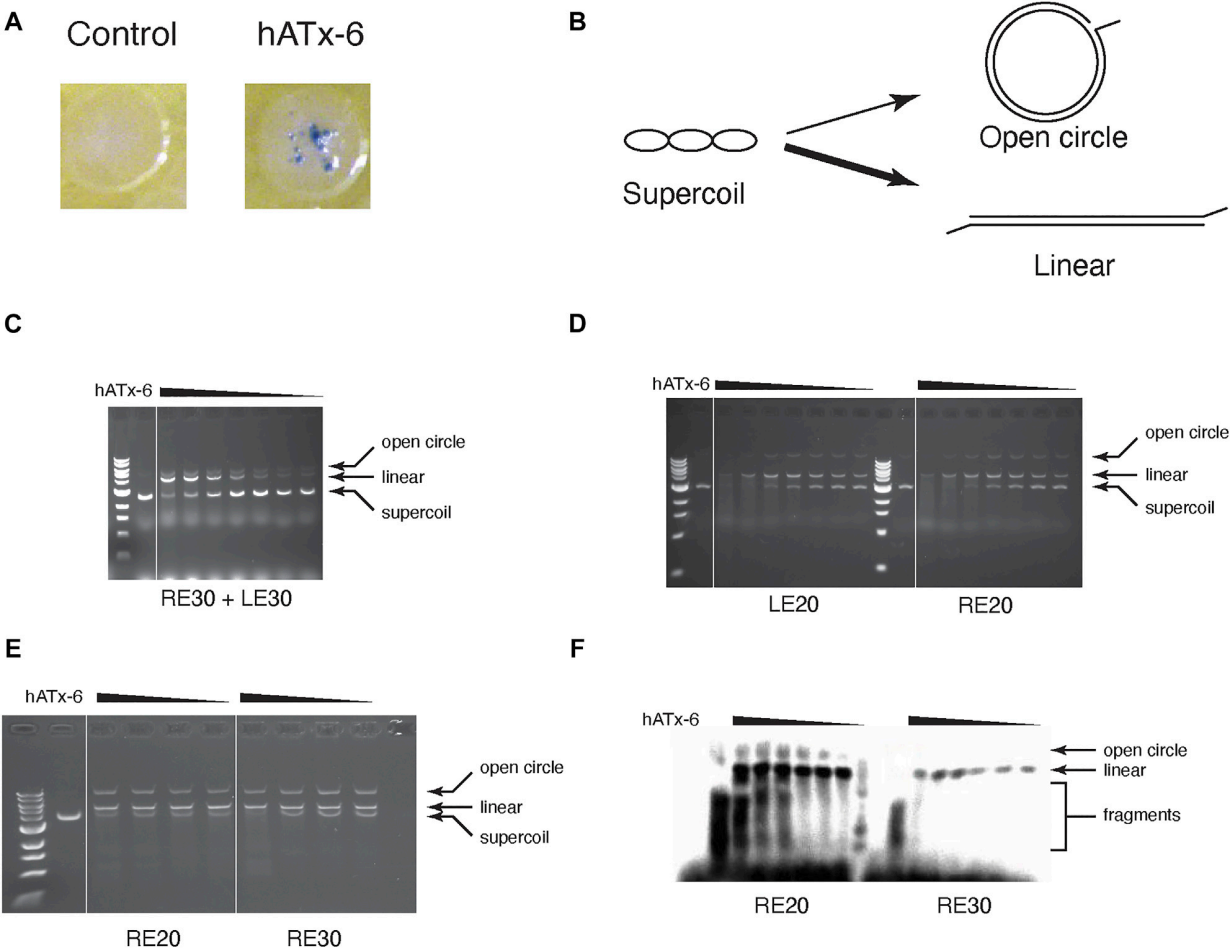
Transposase activity assay of hATx-6. **(A)** Papillation assay of hATx-6 to test transposition activity *in vivo*. Image of a single *E. coli* colony expressing pMAL-c5x (empty vector control) or pMAL-c5X-hATx-6 (hATx-6) grown in rich media with X-gal **(B)** Schematic representation of *in vitro* transposase activity using a supercoiled plasmid DNA. A single nick by a transposase will result in an open circle, while double-strand cleavage or multiple nicking events will yield linearized plasmid DNA. The thickness of the arrows represents that the linearized DNA is a more frequent outcome in these reactions. **(C-E)** Transposition assay of titrated concentrations of transposase (46, 36.8, 27.6, 18.4, and 9.2 μg) with a DNA plasmid and 1 μM of various combinations of transposon ends. **(C)** The reaction contained equal concentrations of the 30 bp right end (RE) and the 30 bp left end (LE). **(D)** Transposase reaction contained either the 20 bp left end (LE) or the 20 bp right end (RE). **(E)** The reaction contained either 20 bp right end (RE) or the 30 bp right end (RE). **(F)** The same reaction conditions as E except that the transposon ends were labelled with Biotin and then imaged using chemiluminescence.

To test the activity of hATx-6 *in vivo*, we performed a papillation assay (Foster et al., 1981) with hATx-6 (Figure 5A). In this assay, a transposon (located on a donor plasmid) contains a *lacZ* gene lacking a promoter. When transposition occurs, there is a chance that the integration site will be downstream of an endogenous promoter in the host genome, leading to expression of *lacZ* gene. Expression of LacZ will cause blue colouration in those cells, due to the presence of X-gal in the agar medium. The number of individual papillae on each colony is indicative of the transposition frequency, it can be clearly seen that the hATx-6 transposase demonstrates multiple transposition events per colony. This was incouriging as it indicates that the hATx-6 transposase is expressed, folds into its native form and is active against the chromosome of *E. coli*.

We tested the purified hATx-6 transposase for activity *in vitro* by using it to cleave supercoiled plasmid in the presence of 20 or 30 base-pair left and right ends of the hATx-6 TE (Figures 5B–F). The activity was not significantly different if we added only the right end or only the left end (Figure 5D), and the shorter oligonucleotide gave reproducibly higher activity than the longer one (Figure 5E). In order to test whether the transposase was joining the TE ends to the plasmid DNA, we labelled the end with biotin and blotted the gel to a nylon membrane and probed with streptavidin-HRP to visualize the reaction products (Figure 5F). These results indicated that both single-end joining and double end joining reactions were occurring and could be detected in these simple *in vitro* reactions.

### Sequence Bias of hATx-6 Transposase

To assess the bias of the hATx-6 transposase in an *in vitro* reaction, we used the transposase to fragment genomic DNA from *E. coli* (51% GC), *Haemophilus influenzae* (38% GC), *Rhodopseudomonas palustris* (65% GC) and human (41% GC) (Figure 4). The reaction stalled at an average fragment size of approximately 2 kb. We then took these fragments and used them to prepare an Illumina library using the Ultra II FS kit. Sequencing results were uploaded to the NCBI SRA database and can be accessed with PRJNA762372 (https://www.ncbi.nlm.nih.gov/bioproject/PRJNA762372). The resulting samples were sequenced on a MiSeq using the paired-end protocol, and the reads were analyzed as follows. We first used the program Cutadapt to identify and isolate reads with the 20 base-pair TE end. About one third of the reads fell in this category. We then combined the two sets of reads produced from the paired-end protocol and created a position weight matrix from the first 28 bases of each read, the first 8 being the TSD and the remaining being the 20 flanking bases (Figure 4A). In the TSD, base 1 showed a moderate G bias, bases 3 and 8 have a moderate C bias, and bases 6 and 7 have a strong A bias. In the flanking region, base 2 has a moderate C bias and base 3 has a strong A bias. Comparing these to the same type of analysis of the insertions in the *Hydra* genome (Figure 4B) gave qualitatively the same result.

**FIGURE 6:**
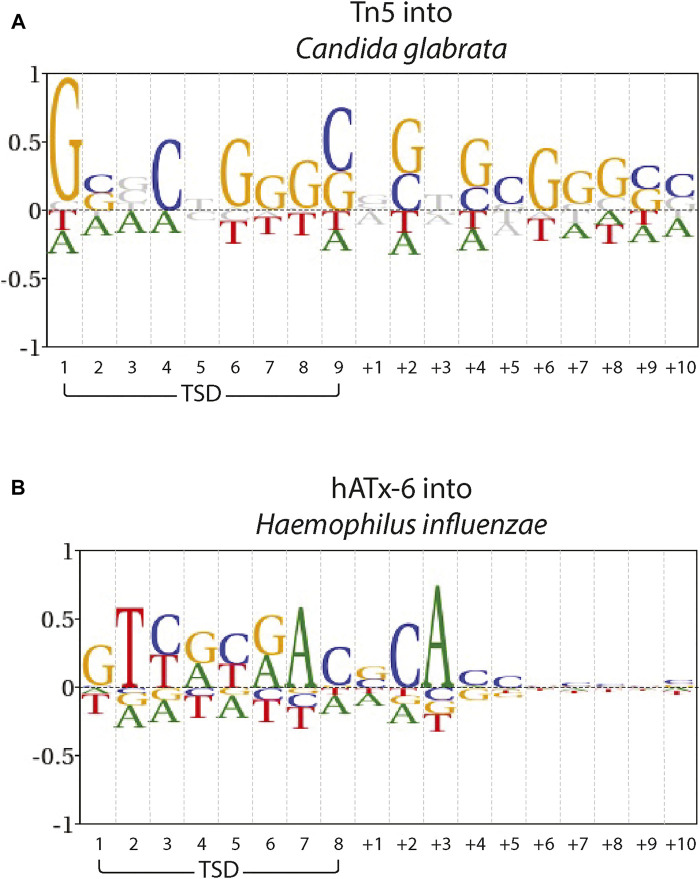
Target site preferences of Tn5 and hAtx-6 transposases. Blogo sequence logos of **(A)** Tn5 into C. glabrata subtelomeric DNA and **(B)** hATx-6 into H. influenzae DNA. The Target Site Duplication (TSD) and the flanking 10 bases as ‘+*x*’ are indicated. Base frequencies are shown compared with the overall DNA content of **(A)** C. glabrata A: 0.32, C: 0.19, G: 0.18, T:0.31 and **(B)** H. influenzae A: 0.31, C: 0.19, G: 0.19, T: 0.31.

To assess whether there was a measurable difference between Tn5 and hATx-6 target site bias, we ran Blogo sequence logos of a previously published Tn5 data set (Green et al., 2012) in *Candida glabrata* subtelomeric DNA, and the hATx-6 data set in *Haemophilus influenzae* DNA (Figure 6). These included the target site duplication region and the flanking 10 bases, and background frequencies were adjusted for the genomic DNA used. The Tn5 insertion site shows GC bias across both the TSD region and the flanking 10 bases. hATx-6 shows no significant GC bias across both the TSD region or the flanking 10 bases. Some individual bases display strongy (over 0.5 bits) bias, in particular sites 2 and 7 of the TSD region and base +3 of the flanking DNA. There is also some purine or pyrimidine selection in the hATx-6 TSD, sites 3–6, (sites 3 and 5 are pyrimidine biased, sites 4 and 6 are purine biased). The bias observed in the TSD and flanking DNA for hATx-6 varies between the four bases, indicating that there is not a significant GC or AT bias in the target site. In summary, the *in silico* predicted low insertion site seqeunce bias of the putative hATx-6 transposase was replicated both *in vivo* and *in vitro*.

## Discussion

For most applications in molecular biology, the ideal transposase is one that has minimal bias at the target site. In the Tn5 system, the most commonly used for applications, there is a general GC bias in its target binding region and flanking DNA (Green et al., 2012). The ITm/mariner systems (such as ISY100 or Sleeping Beauty) there is an absolute bias for a TA dinucleotide TSD, but fairly limited sequence bias in the flanking regions (Feng and Colloms, 2007; Ivics and Izsvák, 2015). Many hAT superfamily members, such as Hermes, show strong bias at specific sites in the TSD region. Hermes has a strong bias for T at base 2 and A at base 7 of the 8 base TSD, while the other 6 bases all show strong bias for 2 bases (Gangadharan et al., 2010). All of these transposases suffer from some target site biases, which inhibit the range of applications for which they would be applicable. hATx-6 does show some target site preference, more than expected based on the initial bioinformatics. However, when compared with Tn5 (Figure 6), the sequence bias overall does not indicate a significant bias towards GC or AT rich genomes. There is also much lower bias in the flanking DNA. Taken together, these results indicate that hATx-6 would be preferable for *in vitro* applications, and in particular for genomes with low GC content.

While what we have shown here is that the hATx-6 transposase is functional and has low target site preference, characteristics of the enzyme could be improved. Screening a mutant library using papillation assay could be used to further enhance its activity, as has been done in other systems (Zhou and Reznikoff, 1997; Mátés et al., 2009; Voigt et al., 2016). The X-ray crystal structure of the hAT transposases Hermes has shown it to exist as octamers, consisting of four dimeric transposase units (Hickman et al., 2014). It is highly likely that the hATx-6 transposase has a similar structure, though further studies to determine this would be of interest. Structural clues might lead to mechanistic details in explaining the low insertion sequence site specificifities of hATx-6. Expression in *E. coli* has a tendency to produce some truncated forms of the protein, which might combine with full-length protein in the octamer and reduce or eliminate its activity, in a similar manner to the inhibitor protein of Tn5 (Braam et al., 1999). Modifications of the growth conditions, application of different host strains, or mutagenesis might improve the production of full-length, active protein. Mutagenesis could also be applied to optimize the solubility, as at present a portion of the protein produced is insoluble. Overcoming these difficulties would require additional work but given the favorable characteristics of the hATx-6 transposase, would be worthwhile.

Identification of new TEs by the examination of target site preference is a methodology not used previously to screen new elements. This approach is a useful methodology for identifying new transposases aimed at particular applications. Instead of modifying a TE to fit an application to which it may not be suited, we might be able to identify the TE optimal for a specific purpose.

## Data Availability

The original contributions presented in the study are publicly available in NCBI https://www.ncbi.nlm.nih.gov/bioproject/762372 under accession number PRJNA762372.
