# Supplementary material for: Insertion Specificity of the hATx-6 Transposase of Hydra magnipapillata
Source: Front Mol Biosci. 2021 Dec 20;8:734154. doi: 10.3389/fmolb.2021.734154 (PMC8721813; doi:10.3389/fmolb.2021.734154)
Supplement: Supplementary file 1 [file Table1.DOCX]

**Supplementary Figure S2: Plasmid Sequences:**

The hATx-6 transposase is highlighted in green. The hATx-6 RE30 is marked in red and the hATx-6 LE30 is marked in blue.

pMAL-c5X-hATx-6:

CCGACACCATCGAATGGTGCAAAACCTTTCGCGGTATGGCATGATAGCGCCCGGAAGAGAGTCAATTCAGGGTGGTGAATGTGAAACCAGTAACGTTATACGATGTCGCAGAGTATGCCGGTGTCTCTTATCAGACCGTTTCCCGCGTGGTGAACCAGGCCAGCCACGTTTCTGCGAAAACGCGGGAAAAAGTGGAAGCGGCGATGGCGGAGCTGAATTACATTCCCAACCGCGTGGCACAACAACTGGCGGGCAAACAGTCGTTGCTGATTGGCGTTGCCACCTCCAGTCTGGCCCTGCACGCGCCGTCGCAAATTGTCGCGGCGATTAAATCTCGCGCCGATCAACTGGGTGCCAGCGTGGTGGTGTCGATGGTAGAACGAAGCGGCGTCGAAGCCTGTAAAGCGGCGGTGCACAATCTTCTCGCGCAACGCGTCAGTGGGCTGATCATTAACTATCCGCTGGATGACCAGGATGCCATTGCTGTGGAAGCTGCCTGCACTAATGTTCCGGCGTTATTTCTTGATGTCTCTGACCAGACACCCATCAACAGTATTATTTTCTCCCATGAAGACGGTACGCGACTGGGCGTGGAGCATCTGGTCGCATTGGGTCACCAGCAAATCGCGCTGTTAGCGGGCCCATTAAGTTCTGTCTCGGCGCGTCTGCGTCTGGCTGGCTGGCATAAATATCTCACTCGCAATCAAATTCAGCCGATAGCGGAACGGGAAGGCGACTGGAGTGCCATGTCCGGTTTTCAACAAACCATGCAAATGCTGAATGAGGGCATCGTTCCCACTGCGATGCTGGTTGCCAACGATCAGATGGCGCTGGGCGCAATGCGCGCCATTACCGAGTCCGGGCTGCGCGTTGGTGCGGATATTTCGGTAGTGGGATACGACGATACCGAAGACAGCTCATGTTATATCCCGCCGTTAACCACCATCAAACAGGATTTTCGCCTGCTGGGGCAAACCAGCGTGGACCGCTTGCTGCAACTCTCTCAGGGCCAGGCGGTGAAGGGCAATCAGCTGTTGCCCGTCTCACTGGTGAAAAGAAAAACCACCCTGGCGCCCAATACGCAAACCGCCTCTCCCCGCGCGTTGGCCGATTCATTAATGCAGCTGGCACGACAGGTTTCCCGACTGGAAAGCGGGCAGTGAGCGCAACGCAATTAATGTAAGTTAGCTCACTCATTAGGCACAATTCTCATGTTTGACAGCTTATCATCGACTGCACGGTGCACCAATGCTTCTGGCGTCAGGCAGCCATCGGAAGCTGTGGTATGGCTGTGCAGGTCGTAAATCACTGCATAATTCGTGTCGCTCAAGGCGCACTCCCGTTCTGGATAATGTTTTTTGCGCCGACATCATAACGGTTCTGGCAAATATTCTGAAATGAGCTGTTGACAATTAATCATCGGCTCGTATAATGTGTGGAATTGTGAGCGGATAACAATTTCACACAGGAAACAGCCAGTCCGTTTAGGTGTTTTCACGAGCAATTGACCAACAAGGACCATAGATTATGAAAATCGAAGAAGGTAAACTGGTAATCTGGATTAACGGCGATAAAGGCTATAACGGTCTCGCTGAAGTCGGTAAGAAATTCGAGAAAGATACCGGAATTAAAGTCACCGTTGAGCATCCGGATAAACTGGAAGAGAAATTCCCACAGGTTGCGGCAACTGGCGATGGCCCTGACATTATCTTCTGGGCACACGACCGCTTTGGTGGCTACGCTCAATCTGGCCTGTTGGCTGAAATCACCCCGGACAAAGCGTTCCAGGACAAGCTGTATCCGTTTACCTGGGATGCCGTACGTTACAACGGCAAGCTGATTGCTTACCCGATCGCTGTTGAAGCGTTATCGCTGATTTATAACAAAGATCTGCTGCCGAACCCGCCAAAAACCTGGGAAGAGATCCCGGCGCTGGATAAAGAACTGAAAGCGAAAGGTAAGAGCGCGCTGATGTTCAACCTGCAAGAACCGTACTTCACCTGGCCGCTGATTGCTGCTGACGGGGGTTATGCGTTCAAGTATGAAAACGGCAAGTACGACATTAAAGACGTGGGCGTGGATAACGCTGGCGCGAAAGCGGGTCTGACCTTCCTGGTTGACCTGATTAAAAACAAACACATGAATGCAGACACCGATTACTCCATCGCAGAAGCTGCCTTTAATAAAGGCGAAACAGCGATGACCATCAACGGCCCGTGGGCATGGTCCAACATCGACACCAGCAAAGTGAATTATGGTGTAACGGTACTGCCGACCTTCAAGGGTCAACCATCCAAACCGTTCGTTGGCGTGCTGAGCGCAGGTATTAACGCCGCCAGTCCGAACAAAGAGCTGGCAAAAGAGTTCCTCGAAAACTATCTGCTGACTGATGAAGGTCTGGAAGCGGTTAATAAAGACAAACCGCTGGGTGCCGTAGCGCTGAAGTCTTACGAGGAAGAGTTGGTGAAAGATCCGCGTATTGCCGCCACTATGGAAAACGCCCAGAAAGGTGAAATCATGCCGAACATCCCGCAGATGTCCGCTTTCTGGTATGCCGTGCGTACTGCGGTGATCAACGCCGCCAGCGGTCGTCAGACTGTCGATGAAGCCCTGAAAGACGCGCAGACTAATTCGAGCTCGAACAACAACAACAATAACAATAACAACAACCTCGGGATCGAGGGAAGGATGAACGAAAATTCTGAAAAGGGCACTAGTTTTTTTCTGCACAAAAAAGATAAAGACCCGACGTCTGTATGGTCTCATTATTTAAAATCTGCGGACAATTTAGCTGCGAAATGTAAGAAGTGCGGGGTTATCCTGAAAACCTTAGGTGGCAGCACCAAAGGCTTGCACACGCACCTCAGCACGAAACACAACCTGAAAGTAACCAGCGCAGACAAGAACCTTCCTTCCTCCTCGCAGCAGCTTAACGACGCACGTACAGACGAGAAACCTCCAACAAAACGCCGCATCACAAGCTATTTCGCTTCGGAAAAAAAAAAAACCCTGGATGAAACCTTAGCACGCATGACCGCATTGGATGGCTTGACGTTTAACGTTTTCATTACCTCAAAAGACCTGCGCCAACTCCTTATTACGTCCGGGTTCGAAAACTTGCCAAAAAGTGCCAATTCGATTAAACGTATTGTGGTGTCTTACTCGCTGAATATCCGTCAGCTCATTAGCAAGGAAATCTATCAATACAAAGCACTGGGCACCCGCTTCAGCTTGTCTTTTGATGAGTGGACGAGCACGACAAACAAGCGCTACATGAACATTAATGTCCACATTCCGAACAAACATTGGAATCTGGGCCTTATCCGTGTCTATGGTTCCATGAACGCGGAGAAGTGCATCGATGTGTTGAAAACCCGTCTTAAAGAACACAACGTTAACATTGATACCGACATCGTGGCCATTGTCACCGACGGCCCGAACGTAATGGTGCGCGTGGGCAAAATTATCGAAGCCGAGCACCAACTGTGTCTGGTGCATGGTATTCATCTGGCCGTTTGTGATGTCTTGTACAAGAAATCAAGCACCCCGATGAGCGATACGGAGGCTCCGCTGAAAGAAACGCAGGATATTGTCGACGAAACGGACGACCATGAAGATGAAGAAAACATCGATGAAGAGATGGGTCTGGGGTTCTTGCCCCCTACCAATGACGGTATTGCTAATATGGACCTTCTGACCAACGAACAAAACATCAATGAACTCGTAAAAAAAGCCCGTAAAGTGGTGGTGCTGTTTAAACGCAGTCCTACGAAAAATGACGCAGTTCTGCAAAAATATGTGAAGGAAGAAAAAGGTAGCGAGCTGTCCTTAATTTTAGACTGCAAAACTCGTTGGAATAGTCTTCTCTCGATGTTAGAACGTTTTCTGGCCCTGAAAACATGCATCCAAAAAGCGTTGATTGATCTCAACCATCCGGTCTCACTGAATGAGAGTGACTTTGTGGTTCTGTCGGAAATCATCGAGGTGCTGGCCCCGGTTAAACTTGCCGTAGATGCCTTGTGCCGTCGCGACGCGAACCTTTGTACTGCCGATGCGGTGCTTATCTTCTTGTTTCAGCAAATCGCCCAGAAACAGTCGCCGTTTGCTAACAAAATGTTCCAGGCGATTAAGACGCGCATGGAAAAACGCCGTTGTGAAGAAATTTCGGGCGTCCTGAAATATTTGCAAAACCCAAAGTACGATGCGGTAAACGATGAAACCAACGTGTGTAAAATCTTCGGAGTGCCGCCGAAAACGCTGATCCGTAAACAAATTAAAAAACTGATTGAACGCCTGGCCGCCTTTAAGAATGATAACCTCGAAAAGTCGTCAATTGATGTTGACGAAAACATCATGGCGGTTGTGCGTAACGAAGAGTCGCAGGTGCGTGCTAGCTTGTCATTGCGCGAAAAGTTACAGGAGCAGATTGCTCAGAGTATGAAAGCGATTGAACCGGAAACCCAGACGTCAAACCTCATGAGCATCATCAAAAAAGAGATGCATCTTTTTGAAAACGGCGGTAACCGCGGCCACAATCTGGAACTGGTTTATCAGTATTTGCTGTCTATCCCGCCAACGAGTATTGAGCCGGAACGCGCCTTCAGCGCGGCTGGCTATTTGGGGTCCAAAATCCGTTCGCGTTTAAATGACCAGACGCTGAACGCACTCTTACTGCTGCGCGCATACTTCCAGACGAAAAAAGAATCTCTGTGATCAAATAAAACGAAAGGCTCAGTCGAAAGACTGGGCCTTTCGTTTTATCTGTTGTTTGTCGGTGAACGCTCTCCTGAGTAGGACAAATCCGCCGGGAGCGGATTTGAACGTTGCGAAGCAACGGCCCGGAGGGTGGCGGGCAGGACGCCCGCCATAAACTGCCAGGCATCAAATTAAGCAGAAGGCCATCCTGACGGATGGCCTTTTTGCGTTTCTACAAACTCTTTCGGTCCGTTGTTTATTTTTCTAAATACATTCAAATATGTATCCGCTCATGAGACAATAACCCTGATAAATGCTTCAATAATATTGAAAAAGGAAGAGTATGAGTATTCAACATTTCCGTGTCGCCCTTATTCCCTTTTTTGCGGCATTTTGCCTTCCTGTTTTTGCTCACCCAGAAACGCTGGTGAAAGTAAAAGATGCTGAAGATCAGTTGGGTGCACGAGTGGGTTACATCGAACTGGATCTCAACAGCGGTAAGATCCTTGAGAGTTTTCGCCCCGAAGAACGTTTCCCAATGATGAGCACTTTTAAAGTTCTGCTATGTGGCGCGGTATTATCCCGTGTTGACGCCGGGCAAGAGCAACTCGGTCGCCgCATACACTATTCTCAGAATGACTTGGTTGAGTACTCACCAGTCACAGAAAAGCATCTTACGGATGGCATGACAGTAAGAGAATTATGCAGTGCTGCCATAACCATGAGTGATAACACTGCGGCCAACTTACTTCTGACAACGATCGGAGGACCGAAGGAGCTAACCGCTTTTTTGCACAACATGGGGGATCATGTAACTCGCCTTGATCGTTGGGAACCGGAGCTGAATGAAGCCATACCAAACGACGAGCGTGACACCACGATGCCTGTAGCAATGGCAACAACGTTGCGCAAACTATTAACTGGCGAACTACTTACTCTAGCTTCCCGGCAACAATTAATAGACTGGATGGAGGCGGATAAAGTTGCAGGACCACTTCTGCGCTCGGCCCTTCCGGCTGGCTGGTTTATTGCTGATAAATCTGGAGCCGGTGAGCGTGGGTCTCGCGGTATCATTGCAGCACTGGGGCCAGATGGTAAGCCCTCCCGTATCGTAGTTATCTACACGACGGGGAGTCAGGCAACTATGGATGAACGAAATAGACAGATCGCTGAGATAGGTGCCTCACTGATTAAGCATTGGTAACTGTCAGACCAAGTTTACTCATATATACTTTAGATTGATTTCCTTAGGACTGAGCGTCAACCCCGTAGAAAAGATCAAAGGATCTTCTTGAGATCCTTTTTTTCTGCGCGTAATCTGCTGCTTGCAAACAAAAAAACCACCGCTACCAGCGGTGGTTTGTTTGCCGGATCAAGAGCTACCAACTCTTTTTCCGAAGGTAACTGGCTTCAGCAGAGCGCAGATACCAAATACTGTCCTTCTAGTGTAGCCGTAGTTAGGCCACCACTTCAAGAACTCTGTAGCACCGCCTACATACCTCGCTCTGCTAATCCTGTTACCAGTGGCTGCTGCCAGTGGCGATAAGTCGTGTCTTACCGGGTTGGACTCAAGACGATAGTTACCGGATAAGGCGCAGCGGTCGGGCTGAACGGGGGGTTCGTGCACACAGCCCAGCTTGGAGCGAACGACCTACACCGAACTGAGATACCTACAGCGTGAGCTATGAGAAAGCGCCACGCTTCCCGAAGGGAGAAAGGCGGACAGGTATCCGGTAAGCGGCAGGGTCGGAACAGGAGAGCGCACGAGGGAGCTTCCAGGGGGAAACGCCTGGTATCTTTATAGTCCTGTCGGGTTTCGCCACCTCTGACTTGAGCGTCGATTTTTGTGATGCTCGTCAGGGGGGCGGAGCCTATGGAAAAACGCCAGCAACGCGGCCTTTTTACGGTTCCTGGCCTTTTGCTGGCCTTTTGCTCACATGTTCTTTCCTGCGTTATCCCCTGATTCTGTGGATAACCGTATTACCGCCTTTGAGTGAGCTGATACCGCTCGCCGCAGCCGAACGACCGAGCGCAGCGAGTCAGTGAGCGAGGAAGCGGAAGAGCGCCTGATGCGGTATTTTCTCCTTACGCATCTGTGCGGTATTTCACACCGCATATAAGGTGCACTGTGACTGGGTCATGGCTGCGCCCCGACACCCGCCAACACCCGCTGACGCGCCCTGACGGGCTTGTCTGCTCCCGGCATCCGCTTACAGACAAGCTGTGACCGTCTCCGGGAGCTGCATGTGTCAGAGGTTTTCACCGTCATCACCGAAACGCGCGAGGCAGCTGCGGTAAAGCTCATCAGCGTGGTCGTGCAGCGATTCACAGATGTCTGCCTGTTCATCCGCGTCCAGCTCGTTGAGTTTCTCCAGAAGCGTTAATGTCTGGCTTCTGATAAAGCGGGCCATGTTAAGGGCGGTTTTTTCCTGTTTGGTCACTGATGCCTCCGTGTAAGGGGGATTTCTGTTCATGGGGGTAATGATACCGATGAAACGAGAGAGGATGCTCACGATACGGGTTACTGATGATGAACATGCCCGGTTACTGGAACGTTGTGAGGGTAAACAACTGGCGGTATGGATGCGGCGGGACCAGAGAAAAATCACTCAGGGTCAATGCCAGCGCTTCGTTAATACAGATGTAGGTGTTCCACAGGGTAGCCAGCAGCATCCTGCGATGCAGATCCGGAACATAATGGTGCAGGGCGCTGACTTCCGCGTTTCCAGACTTTACGAAACACGGAAACCGAAGACCATTCATGTTGTTGCTCAGGTCGCAGACGTTTTGCAGCAGCAGTCGCTTCACGTTCGCTCGCGTATCGGTGATTCATTCTGCTAACCAGTAAGGCAACCCCGCCAGCCTAGCCGGGTCCTCAACGACAGGAGCACGATCATGCGCACCCGTGGCCAGGACCCAACGCTGCCCGAAATT

pSYX-RE30-lacZ-LE30:

TAGGGATTGCAATCCCGGATCCCGAGATCCCCTGCAGGCTCGAGTTATTATTATTTTTGACACCAGACCAACTGGTAATGGTAGCGACCGGCGCTCAGCTGGAATTCCGCCGATACTGACGGGCTCCAGGAGTCGTCGCCACCAATCCCCATATGGAAACCGTCGATATTCAGCCATGTGCCTTCTTCCGCGTGCAGCAGATGGCGATGGCTGGTTTCCATCAGTTGCTGTTGACTGTAGCGGCTGATGTTGAACTGGAAGTCGCCGCGCCACTGGTGTGGGCCATAATTCAATTCGCGCGTCCCGCAGCGCAGACCGTTTTCGCTCGGGAAGACGTACGGGGTATACATGTCTGACAATGGCAGATCCCAGCGGTCAAAACAGGCGGCAGTAAGGCGGTCGGGATAGTTTTCTTGCGGCCCTAATCCGAGCCAGTTTACCCGCTCTGCTACCTGCGCCAGCTGGCAGTTCAGGCCAATCCGCGCCGGATGCGGTGTATCGCTCGCCACTTCAACATCAACGGTAATCGCCATTTGACCACTACCATCAATCCGGTAGGTTTTCCGGCTGATAAATAAGGTTTTCCCCTGATGCTGCCACGCGTGAGCGGTCGTAATCAGCACCGCATCAGCAAGTGTATCTGCCGTGCACTGCAACAACGCTGCTTCGGCCTGGTAATGGCCCGCCGCCTTCCAGCGTTCGACCCAGGCGTTAGGGTCAATGCGGGTCGCTTCACTTACGCCAATGTCGTTATCCAGCGGTGCACGGGTGAACTGATCGCGCAGCGGCGTCAGCAGTTGTTTTTTATCGCCAATCCACATCTGTGAAAGAAAGCCTGACTGGCGGTTAAATTGCCAACGCTTATTACCCAGCTCGATGCAAAAATCCATTTCGCTGGTGGTCAGATGCGGGATGGCGTGGGACGCGGCGGGGAGCGTCACACTGAGGTTTTCCGCCAGACGCCACTGCTGCCAGGCGCTGATGTGCCCGGCTTCTGACCATGCGGTCGCGTTCGGTTGCACTACGCGTACTGTGAGCCAGAGTTGCCCGGCGCTCTCCGGCTGCGGTAGTTCAGGCAGTTCAATCAACTGTTTACCTTGTGGAGCGACATCCAGAGGCACTTCACCGCTTGCCAGCGGCTTACCATCCAGCGCCACCATCCAGTGCAGGAGCTCGTTATCGCTATGACGGAACAGGTATTCGCTGGTCACTTCGATGGTTTGCCCGGATAAACGGAACTGGAAAAACTGCTGCTGGTGTTTTGCTTCCGTCAGCGCTGGATGCGGCGTGCGGTCGGCAAAGACCAGACCGTTCATACAGAACTGGCGATCGTTCGGCGTATCGCCAAAATCACCGCCGTAAGCCGACCACGGGTTGCCGTTTTCATCATATTTAATCAGCGACTGATCCACCCAGTCCCAGACGAAGCCGCCCTGTAAACGGGGATACTGACGAAACGCCTGCCAGTATTTAGCGAAACCGCCAAGACTGTTACCCATCGCGTGGGCGTATTCGCAAAGGATCAGCGGGCGCGTCTCTCCAGGTAGCGAAAGCCATTTTTTGATGGACCATTTCGGCACAGCCGGGAAGGGCTGGTCTTCATCCACGCGCGCGTACATCGGGCAAATAATATCGGTGGCCGTGGTGTCGGCTCCGCCGCCTTCATACTGCACCGGGCGGGAAGGATCGACAGATTTGATCCAGCGATACAGCGCGTCGTGATTAGCGCCGTGGCCTGATTCATTCCCCAGCGACCAGATGATCACACTCGGGTGATTACGATCGCGCTGCACCATTCGCGTTACGCGTTCGCTCATCGCCGGTAGCCAGCGCGGATCATCGGTCAGACGATTCATTGGCACCATGCCGTGGGTTTCAATATTGGCTTCATCCACCACATACAGGCCGTAGCGGTCGCACAGCGTGTACCACAGCGGATGGTTCGGATAATGCGAACAGCGCACGGCGTTAAAGTTGTTCTGCTTCATCAGCAGGATATCCTGCACCATCGTCTGCTCATCCATGACCTGACCATGCAGAGGATGATGCTCGTGACGGTTAACGCCTCGAATCAGCAACGGCTTGCCGTTCAGCAGCAGCAGACCATTTTCAATCCGCACCTCGCGGAAACCGACATCGCAGGCTTCTGCTTCAATCAGCGTGCCGTCGGCGGTGTGCAGTTCAACCACCGCACGATAGAGATTCGGGATTTCGGCGCTCCACAGTTTCGGGTTTTCGACGTTCAGACGTAGTGTGACGCGATCGGCATAACCACCACGCTCATCGATAATTTCACCGCCGAAAGGCGCGGTGCCGCTGGCGACCTGCGTTTCACCCTGCCATAAAGAAACTGTTACCCGTAGGTAGTCACGCAACTCGCCGCACATCTGAACTTCAGCCTCCAGTACAGCGCGGCTGAAATCATCATTAAAGCGAGTGGCAACATGGAAATCGCTGATTTGTGTAGTCGGTTTATGCAGCAACGAGACGTCACGGAAAATGCCGCTCATCCGCCACATATCCTGATCTTCCAGATAACTGCCGTCACTCCAACGCAGCACCATCACCGCGAGGCGGTTTTCTCCGGCGCGTAAAAATGCGCTCAGGTCAAATTCAGACGGCAAACGACTGTCCTGGCCGTAACCGACCCAGCGCCCGTTGCACCACAGATGAAACGCCGAGTTAACGCCATCAAAAATAATTCGCGTCTGGCCTTCCTGTAGCCAGCTTTCATCAACATTAAATGTGAGCGAGTAACAACCCGTCGGATTCTCCGTGGGAACAAACGGCGGATTGACCGTAATGGGATAGGTTACGTTGGTGTAGATGGGCGCATCGTAACCGTGCATCTGCCAGTTTGAGGGGACGACGACAGTATCGGCCTCAGGAAGATCGCACTCCAGCCAGCTTTCCGGCACCGCTTCTGGTGCCGGAAACCAGGCAAAGCGCCATTCGCCATTCAGGCTGCGCAACTGTTGGGAAGGGCGATCGGTGCGGGCCTCTTCGCTATTACGCCAGCTGGCGAAAGGGGGATGTGCTGCAAGGCGATTAAGTTGGGTAACGCCAGGGTTTTCCCAGTCACGACGTTGTAAAACGACGGCCAGTGAATCCGTAATCATGGTCATCGTAATTCATGCCCGGGATTGCAATCCCTAGCAGCGAACTGAATGTCACGAAAAAGACAGCGACTCAGGTGCCTGATGGTCGGAGACAAAAGGAATATTCAGCGATTTGCCCGAGCTTGCGAGGGTGCTACTTAAGCCTTTAGGGTTTTAAGGTCTGTTTTGTAGAGGAGCAAACAGCGTTTGCGACATCCTTTTGTAATACTGCGGAACTGACTAAAGTAGTGAGTTATACACAGGGCTGGGATCTATTCTTTTTATCTTTTTTTATTCTTTCTTTATTCTATAAATTATAACCACTTGAATATAAACAAAAAAAACACACAAAGGTCTAGCGGAATTTACAGAGGGTCTAGCAGAATTTACAAGTTTTCCAGCAAAGGTCTAGCAGAATTTACAGATACCCACAACTCAAAGGAAAAGGACTAGTAATTATCATTGACTAGCCCATCTCAATTGGTATAGTGATTAAAATCACCTAGACCAATTGAGATGTATGTCTGAATTAGTTGTTTTCAAAGCAAATGAACTAGCGATTAGTCGCTATGACTTAACGGAGCATGAAACCAAGCTAATTTTATGCTGTGTGGCACTACTCAACCCCACGATTGAAAACCCTACAAGGAAAGAACGGACGGTATCGTTCACTTATAACCAATACGCTCAGATGATGAACATCAGTAGGGAAAATGCTTATGGTGTATTAGCTAAAGCAACCAGAGAGCTGATGACGAGAACTGTGGAAATCAGGAATCCTTTGGTTAAAGGCTTTGAGATTTTCCAGTGGACAAACTATGCCAAGTTCTCAAGCGAAAAATTAGAATTAGTTTTTAGTGAAGAGATATTGCCTTATCTTTTCCAGTTAAAAAAATTCATAAAATATAATCTGGAACATGTTAAGTCTTTTGAAAACAAATACTCTATGAGGATTTATGAGTGGTTATTAAAAGAACTAACACAAAAGAAAACTCACAAGGCAAATATAGAGATTAGCCTTGATGAATTTAAGTTCATGTTAATGCTTGAAAATAACTACCATGAGTTTAAAAGGCTTAACCAATGGGTTTTGAAACCAATAAGTAAAGATTTAAACACTTACAGCAATATGAAATTGGTGGTTGATAAGCGAGGCCGCCCGACTGATACGTTGATTTTCCAAGTTGAACTAGATAGACAAATGGATCTCGTAACCGAACTTGAGAACAACCAGATAAAAATGAATGGTGACAAAATACCAACAACCATTACATCAGATTCCTACCTACATAACGGACTAAGAAAAACACTACACGATGCTTTAACTGCAAAAATTCAGCTCACCAGTTTTGAGGCAAAATTTTTGAGTGACATGCAAAGTAAGTATGATCTCAATGGTTCGTTCTCATGGCTCACGCAAAAACAACGAACCACACTAGAGAACATACTGGCTAAATACGGAAGGATCTGAGGTTCTTATGGCTCTTGTATCTATCAGTGAAGCATCAAGACTAACAAACAAAAGTAGAACAACTGTTCACCGTTACATATCAAAGGGAAAACTGTCCATATGCACAGATGAAAACGGTGTAAAAAAGATAGATACATCAGAGCTTTTACGAGTTTTTGGTGCATTCAAAGCTGTTCACCATGAACAGATCGACAATGTAACAGATGAACAGCATGTAACACCTAATAGAACAGGTGAAACCAGTAAAACAAAGCAACTAGAACATGAAATTGAACACCTGAGACAACTTGTTACAGCTCAACAGTCACACATAGACAGCCTGAAACAGGCGATGCTGCTTATCGAATCAAAGCTGCCGACAACACGGGAGCCAGTGACGCCTCCCGTGGGGAAAAAATCATGGCAATTCTGGAAGAAATAGCGCTTTCAGCCGGCAAACCGGCTGAAGCCGGATCTGCGATTCTGATAACAAACTAGCAACACCAGAACAGCCCGTTTGCGGGCAGCAAAACCCGTACTTTTGGACGTTCCGGCGGTTTTTTGTGGCGAGTGGTGTTCGGGCGGTGCGCGATTATTGAAGCATTTATCAGGGTTATTGTCTCATGAGCGGATACATATTTGAATGTATTTAGAAAAATAAACAAATAGGGGTTCCGCGCACATTTCCCCGAAAAGTGCCACCTGACGTCTAAGAAACCATTATTATCATGACATTAACCTATAAAAATAGGCGTATCACGAGGCCCTTTCGTCTTCAAGAATTCTCATGTTTGACAGCTTATCATCGATAAGCTTTAATGCGGTAGTTTATCACAGTTAAATTGCTAACGCAGTCAGGCACCGTGTATGAAATCTAACAATGCGCTCATCGTCATCCTCGGCACCGTCACCCTGGATGCTGTAGGCATAGGCTTGGTTATGCCGGTACTGCCGGGCCTCTTGCGGGATATCGTCCATTCCGACAGCATCGCCAGTCACTATGGCGTGCTGCTAGCGCTATATGCGTTGATGCAATTTCTATGCGCACCCGTTCTCGGAGCACTGTCCGACCGCTTTGGCCGCCGCCCAGTCCTGCTCGCTTCGCTACTTGGAGCCACTATCGACTACGCGATCATGGCGACCACACCCGTCCTGTGGATCCTCTACGCCGGACGCATCGTGGCCGGCATCACCGGCGCCACAGGTGCGGTTGCTGGCGCCTATATCGCCGACATCACCGATGGGGAAGATCGGGCTCGCCACTTCGGGCTCATGAGCGCTTGTTTCGGCGTGGGTATGGTGGCAGGCCCCGTGGCCGGGGGACTGTTGGGCGCCATCTCCTTGCATGCACCATTCCTTGCGGCGGCGGTGCTCAACGGCCTCAACCTACTACTGGGCTGCTTCCTAATGCAGGAATCGCATAAGGGAGAGCGTCGACCGATGCCCTTGAGAGCCTTCAACCCAGTCAGCTCCTTCCGGTGGGCGCGGGGCATGACTATCGTCGCCGCACTTATGACTGTCTTCTTTATCATGCAACTCGTAGGACAGGTGCCGGCAGCGCTCTGGGTCATTTTCGGCGAGGACCGCTTTCGCTGGAGCGCGACGATGATCGGCCTGTCGCTTGCGGTATTCGGAATCTTGCACGCCCTCGCTCAAGCCTTCGTCACTGGTCCCGCCACCAAACGTTTCGGCGAGAAGCAGGCCATTATCGCCGGCATGGCGGCCGACGCGCTGGGCTACGTCTTGCTGGCGTTCGCGACGCGAGGCTGGATGGCCTTCCCCATTATGATTCTTCTCGCTTCCGGCGGCATCGGGATGCCCGCGTTGCAGGCCATGCTGTCCAGGCAGGTAGATGACGACCATCAGGGACAGCTTCAAGGATCGCTCGCGGCTcTTACCAGCCTAACTTCGATCATTGGACCGCTGATCGTCACGGCGATTTATGCCGCCTCGGCGAGCACATGGAACGGGTTGGCATGGATTGTAGGCGCCGCCCTATACCTTGTCTGCCTCCCCGCGTTGCGTCGCGGTGCATGGAGCCGGGCCACCTCGACCTGAATGGAAGCCGGCGGCACCTCGCTGCGTCGTGTAGGGAAAGAGTGTAGATCTAACGGATTCACCACTCCAAGAATTGGAGCCAATCAATTCTTGCGGAGAACTGTGAATGCGCAAAC

**g-blocks for hATx-6**

agcaggaggaattcacATGAACGAAAATTCTGAAAAGGGCACTAGTTTTTTTCTGCACAAAAAAGATAAAGACCCGACGTCTGTATGGTCTCATTATTTAAAATCTGCGGACAATTTAGCTGCGAAATGTAAGAAGTGCGGGGTTATCCTGAAAACCTTAGGTGGCAGCACCAAAGGCTTGCACACGCACCTCAGCACGAAACACAACCTGAAAGTAACCAGCGCAGACAAGAACCTTCCTTCCTCCTCGCAGCAGCTTAACGACGCACGTACAGACGAGAAACCTCCAACAAAACGCCGCATCACAAGCTATTTCGCTTCGGAAAAAAAAAAAACCCTGGATGAAACCTTAGCACGCATGACCGCATTGGATGGCTTGACGTTTAACGTTTTCATTACCTCAAAAGACCTGCGCCAACTCCTTATTACGTCCGGGTTCGAAAACTTGCCAAAAAGTGCCAATTCGATTAAACGTATTGTGGTGTCTTACTCGCTGAATATCCGTCAGCTCATTAGCAAGGAAATCTATCAATACAAAGCACTGGGCACCCGCTTCAGCTTGTCTTTTGATGAGTGGACGAGCACGACAAACAAGCGCTACATGAACATTAATGTCCACATTCCGAACAAACATTGGAATCTGGGCCTTATCCGTGTCTATGGTTCCATGAACGCGGAGAAGTGCATCGATGTGTTGAAAACCCGT/CTTAAAGAACACAACGTTAACATTGATACCGAC

hATx-6.1 11881 1463

CTTAAAGAACACAACGTTAACATTGATACCGACATCGTGGCCATTGTCACCGACGGCCCGAACGTAATGGTGCGCGTGGGCAAAATTATCGAAGCCGAGCACCAACTGTGTCTGGTGCATGGTATTCATCTGGCCGTTTGTGATGTCTTGTACAAGAAATCAAGCACCCCGATGAGCGATACGGAGGCTCCGCTGAAAGAAACGCAGGATATTGTCGACGAAACGGACGACCATGAAGATGAAGAAAACATCGATGAAGAGATGGGTCTGGGGTTCTTGCCCCCTACCAATGACGGTATTGCTAATATGGACCTTCTGACCAACGAACAAAACATCAATGAACTCGTAAAAAAAGCCCGTAAAGTGGTGGTGCTGTTTAAACGCAGTCCTACGAAAAATGACGCAGTTCTGCAAAAATATGTGAAGGAAGAAAAAGGTAGCGAGCTGTCCTTAATTTTAGACTGCAAAACTCGTTGGAATAGTCTTCTCTCGATGTTAGAACGTTTTCTGGCCCTGAAAACATGCATCCAAAAAGCGTTGATTGATCTCAACCATCCGGTCTCACTGAATGAGAGTGACTTTGTGGTTCTGTCGGAAATCATCGAGGTGCTGGCCCC/GGTTAAACTTGCCGTAGATG

hATx-6.2 11881 1464

GGTTAAACTTGCCGTAGATGCCTTGTGCCGTCGCGACGCGAACCTTTGTACTGCCGATGCGGTGCTTATCTTCTTGTTTCAGCAAATCGCCCAGAAACAGTCGCCGTTTGCTAACAAAATGTTCCAGGCGATTAAGACGCGCATGGAAAAACGCCGTTGTGAAGAAATTTCGGGCGTCCTGAAATATTTGCAAAACCCAAAGTACGATGCGGTAAACGATGAAACCAACGTGTGTAAAATCTTCGGAGTGCCGCCGAAAACGCTGATCCGTAAACAAATTAAAAAACTGATTGAACGCCTGGCCGCCTTTAAGAATGATAACCTCGAAAAGTCGTCAATTGATGTTGACGAAAACATCATGGCGGTTGTGCGTAACGAAGAGTCGCAGGTGCGTGCTAGCTTGTCATTGCGCGAAAAGTTACAGGAGCAGATTGCTCAGAGTATGAAAGCGATTGAACCGGAAACCCAGACGTCAAACCTCATGAGCATCATCAAAAAAGAGATGCATCTTTTTGAAAACGGCGGTAACCGCGGCCACAATCTGGAACTGGTTTATCAGTATTTGCTGTCTATCCCGCCAACGAGTATTGAGCCGGAACGCGCCTTCAGCGCGGCTGGCTATTTGGGGTCCAAAATCCGTTCGCGTTTAAATGACCAGACGCTGAACGCACTCTTACTGCTGCGCGCATACTTCCAGACGAAAAAAGAATCTCTGcatcatcatcaccaccact

hATx-6.3 11881 1465
