# Supplementary figures and images for: Insertion Specificity of the hATx-6 Transposase of Hydra magnipapillata
Source: Front Mol Biosci. 2021 Dec 20;8:734154. doi: 10.3389/fmolb.2021.734154 (PMC8721813; doi:10.3389/fmolb.2021.734154)

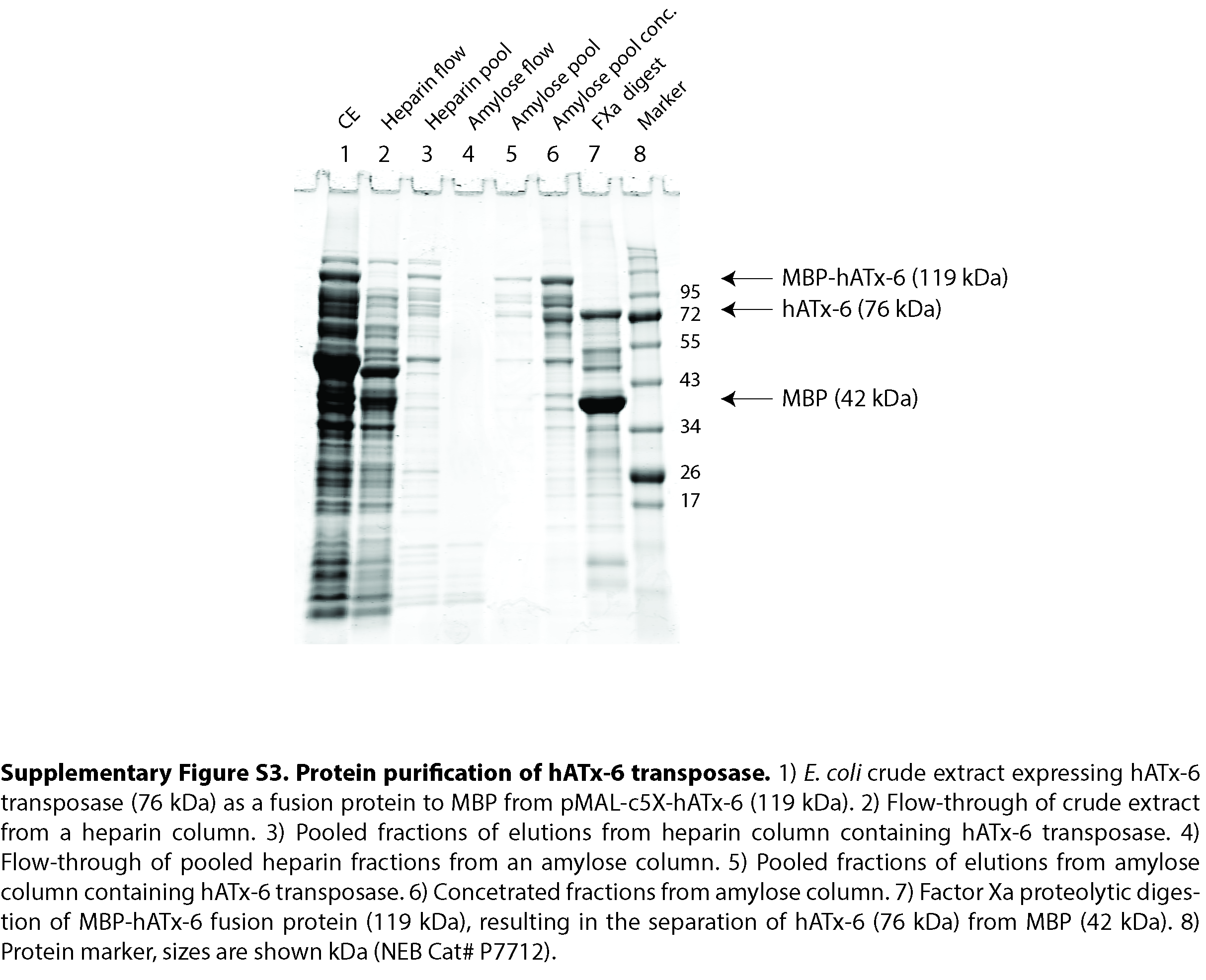

Supplement: Supplementary file 2 [file Image2.TIF]

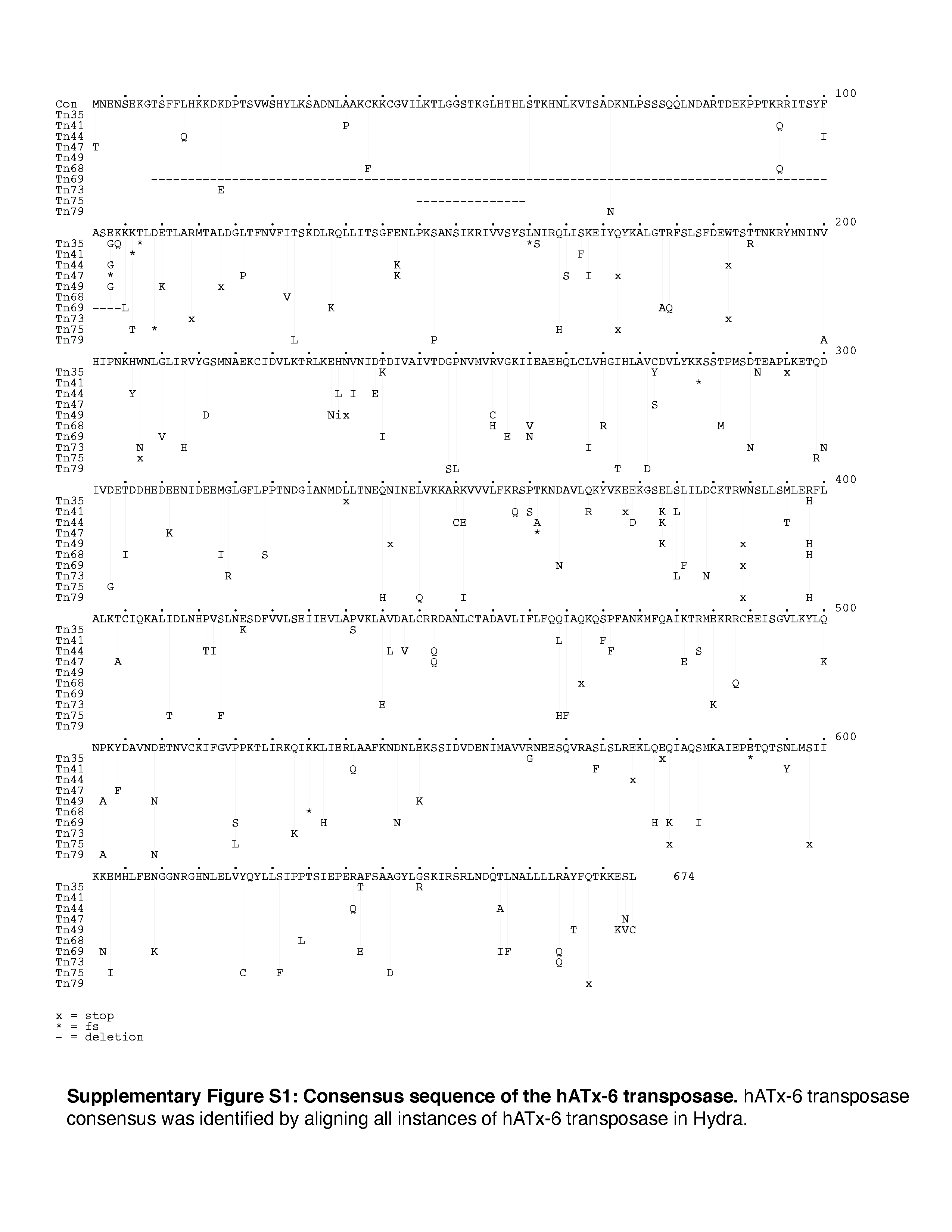

Supplement: Supplementary file 3 [file Image1.TIF]
