# Supplementary material for: Insertion Specificity of the hATx-6 Transposase of Hydra magnipapillata
Source: Front Mol Biosci. 2021 Dec 20;8:734154. doi: 10.3389/fmolb.2021.734154 (PMC8721813; doi:10.3389/fmolb.2021.734154)
Supplement: Supplementary file 4 [file Table2.DOCX]

**Supplementary Table 1: List of scripts used.**

**Script 1: TSD+flank_PWM.py**

#!/usr/bin/python

#creates a PWM from the TSD and 21 base flank of a fastq sequence file

#from Bio import SeqIO

#import re

#import itertools

#import sys, getopt

from Bio.Seq import Seq

from Bio import motifs

from Bio.Alphabet import IUPAC

from Bio.SeqIO.QualityIO import FastqGeneralIterator

sequence = ''

flank = ''

flank_list = []

count = 0

#get sequences, slice first 28 bases (TSD and first 20 of flanking)

#create fasta file and list

with open("ECend_combined.fastq") as seq_records, open("EcTSDflank.fas", "w") as f1:

for title, seq, qual in FastqGeneralIterator(seq_records):

sequence = str(seq)

flank = sequence[0:28]

f1.write(">flank_"+ str(count))

f1.write("\n")

f1.write(str(flank)+ "\n")

flank_list.append(flank)

count += 1

#create text file containing PWM

with open("Ec_pwm.txt", "w") as f1:

motif = motifs.Motif(alphabet=IUPAC.unambiguous_dna)

motif = motifs.create(flank_list)

pwm = motif.counts.normalize(pseudocounts=0.5)

pwm_str = str(pwm)

f1.write(pwm_str)

print 'Number of sequences: ', count

**Script 2: run_blast_return_seq_log_0.4.py**

from operator import itemgetter

from Bio.Blast.Applications import NcbiblastnCommandline

from Bio.Blast import NCBIXML

from Bio import SeqIO

import re

import itertools

import sys, getopt

from Bio.Seq import Seq

from Bio import Motif

from Bio.Alphabet import IUPAC

#usage : python run_blast_return_seq_log.py -i input_file.fa -o logo.png

#this program takes the query sequence (fasta format) and blast it against the genome define in variable genome. The hits are parsed and to be considered as a valid location for a transposon two adjecent hits should :

#[1] be less than 20 kb appart

#[2] first hit is on the + strand and the second hit on the - strand

#[3] the x bp flanking both hits should be direct repeats

#

#the output of the program is a logo file (-o logo.png) of the x bp motif PWM.

#important constance :

size_of_the_direct_repeat = 8

size_of_the_transposon = 30000

genome ="/Users/riggs/Documents/Sequences/Genomes/Hydra/Hydra.fas";

#deals with IO

def mainio(argv):

inputfile = ''

outputfile = ''

try:

opts, args = getopt.getopt(argv,"hi:o:",["ifile=","ofile="])

except getopt.GetoptError:

print 'test.py -i <inputfile> -o <outputfile>'

sys.exit(2)

for opt, arg in opts:

if opt == '-h':

print 'test.py -i <inputfile> -o <outputfile>'

sys.exit()

elif opt in ("-i", "--ifile"):

inputfile = arg

elif opt in ("-o", "--ofile"):

outputfile = arg

return (inputfile, outputfile)

#=================================

def hamming(str1, str2):

return sum(itertools.imap(str.__ne__, str1, str2))

inputfile, outputfile = mainio(sys.argv[1:])

blast_xml_file = "blast_output.xml"

#blast command line

blastn_cline = NcbiblastnCommandline(query=inputfile, db=genome ,outfmt=5, out= blast_xml_file, num_alignments = '10000000', word_size = 5, evalue=30)

stdout, stderr = blastn_cline()

#parse blast output in xml format

result_handle = open(blast_xml_file)

blast_records = NCBIXML.parse(result_handle)

#get_the genome to get the flanking sequence and / or the transposon sequence

genome_handle = open(genome, "rU")

genome_record_dict = SeqIO.to_dict(SeqIO.parse(genome_handle, "fasta"))

#create a motif object (for the PWM)

motif = Motif.Motif(alphabet=IUPAC.unambiguous_dna)

#go over the hits

count =0

for blast_record in blast_records :

for alignment in blast_record.alignments :

title = alignment.title

#subject_id is the contig name (genome)

subject_id = title.split(" ")[1]

subject_id = subject_id.rstrip('\n')

#list of hsp hits for contig (subject_id)

hsp_list = []

for hsp in alignment.hsps:

list1 = [hsp.sbjct_start, hsp.frame, hsp.query_start]

hsp_list.append(list1)

sorted_hsp_list = sorted(hsp_list, key=itemgetter(0))

hsp_list_size = len(sorted_hsp_list)

print hsp_list_size

if hsp_list_size >1:

for i in range (0, hsp_list_size-1):

print i

start1 = sorted_hsp_list[i][0]

start2 = sorted_hsp_list[i+1][0]

strand1 = str(sorted_hsp_list[i][1])

strand2 = str(sorted_hsp_list[i+1][1])

minus = "-"

#IMPORTANT : if two flanking hits are less than 20 kb appart and are in inverse orientation +/-

if (start2 - start1 < size_of_the_transposon and (minus not in strand1 and minus in strand2)):

query_start1 = sorted_hsp_list[i][2]

query_start2 = sorted_hsp_list[i+1][2]

# print query_start1

# print query_start2

# print hsp.sbjct

#get the start and end of the transposon starting from the inverted repeat (leaving out the direct repeats)

#the query start is used to know how off the alignment is from the real start

real_start1 = start1-query_start1

real_start2 = start2+query_start2-1

#sequence

transposon = genome_record_dict[subject_id].seq[real_start1:real_start2]

#=========testing the direct repeats they should be the same or closely related.

flanking_repeat1 = str(genome_record_dict[subject_id].seq[real_start1-size_of_the_direct_repeat:real_start1])

flanking_repeat2 = str(genome_record_dict[subject_id].seq[real_start2:real_start2+size_of_the_direct_repeat])

flanking_repeat2_big = str(genome_record_dict[subject_id].seq[real_start2-2:real_start2+size_of_the_direct_repeat+2])

missmatch = hamming(flanking_repeat1, flanking_repeat2)

print flanking_repeat1

print flanking_repeat2

print flanking_repeat2_big

print missmatch

#IMPORTANT : condition : if the direct repeats are at a hamming distance of 1 (one change) or shifted from no more than 2 bp :

if flanking_repeat1 in flanking_repeat2_big or missmatch < 2 :

real_start1 = start1-query_start1

real_start2 = start2+query_start2-1

#sequence

transposon = genome_record_dict[subject_id].seq[real_start1:real_start2]

count = count+1

id_line = "test"

#outputf.write(id_line)

#outputf.write(transposon)

if len(flanking_repeat1) == size_of_the_direct_repeat : motif.add_instance(Seq(flanking_repeat1,motif.alphabet))

if len(flanking_repeat2) == size_of_the_direct_repeat : motif.add_instance(Seq(flanking_repeat2,motif.alphabet))

#get the logo file if more than x instances of the direct repeat motif

print outputfile

count_i =0

for i in motif.instances:

count_i = count_i +1

print count_i

if count_i > 10:

motif.weblogo(outputfile)

**Script 3: run_blast_return_transposon_new_0.1.py**

from operator import itemgetter

from Bio.Blast.Applications import NcbiblastnCommandline

from Bio.Blast import NCBIXML

from Bio import SeqIO

import re

import sys, getopt

import itertools

from Bio.Seq import Seq

from Bio import Motif

from Bio import motifs

from Bio.Alphabet import IUPAC

genome = "/Users/riggs/Documents/Sequences/Genomes/Hydra/Hydra.fas"

#usage : python run_blast_return_seq_log.py -i input_file.fa -o transposon.fa

#this program takes the query sequence (fasta format) and blast it against the genome define in variable hydra_genome. The hits are parsed and to be considered as a valid location for a transposon two adjecent hits should :

#[1] be less than 20 kb appart

#[2] first hit is on the + strand and the second hit on the - strand

#[3] the size_of_the_direct_repeat bp fanking both hits should be direct repeats

#

#the output of the program is a file containing the transposon (including the inverse repeat) satifying the above criteria.

size_of_the_direct_repeat = 8

size_of_the_transposon = 20000

def hamming(str1, str2):

return sum(itertools.imap(str.__ne__, str1, str2))

#deals with IO

def mainio(argv):

inputfile = ''

outputfile = ''

try:

opts, args = getopt.getopt(argv,"hi:o:",["ifile=","ofile="])

except getopt.GetoptError:

print 'test.py -i <inputfile> -o <outputfile>'

sys.exit(2)

for opt, arg in opts:

if opt == '-h':

print 'test.py -i <inputfile> -o <outputfile>'

sys.exit()

elif opt in ("-i", "--ifile"):

inputfile = arg

elif opt in ("-o", "--ofile"):

outputfile = arg

return (inputfile, outputfile)

#=================================

inputfile, outputfile = mainio(sys.argv[1:])

#open output file

f1 = open(outputfile, 'w+')

blast_xml_file = "blast_output.xml"

#blast command line

blastn_cline = NcbiblastnCommandline(query=inputfile, db=genome ,outfmt=5, out= blast_xml_file, num_alignments = '10000000', word_size = 5, evalue=30)

stdout, stderr = blastn_cline()

#parse blast output in xml format

result_handle = open(blast_xml_file)

blast_records = NCBIXML.parse(result_handle)

#get_the hydra genome to get the flanking sequence and / or the transposon sequence

genome_handle = open(genome, "rU")

genome_record_dict = SeqIO.to_dict(SeqIO.parse(genome_handle, "fasta"))

#get the inverted repeats

records = list(SeqIO.parse(inputfile, "fasta"))

print(records[0].id)

nine_bp_inverted_repeat = records[0].seq[0:9].upper()

nine_bp_inverted_repeat_rc = nine_bp_inverted_repeat.reverse_complement().upper()

length_nine_bp_inverted_repeat = len(nine_bp_inverted_repeat)

print(nine_bp_inverted_repeat_rc)

print(nine_bp_inverted_repeat)

#create a motif object (for the PWM)

motif = Motif.Motif(alphabet=IUPAC.unambiguous_dna)

#go over the hits

count =0

for blast_record in blast_records :

for alignment in blast_record.alignments:

title = alignment.title

#subject_id is the contig name (genome)

subject_id = title.split(" ")[1]

subject_id = subject_id.rstrip('\n')

#list of hsp hits for contig (subject_id)

hsp_list = []

for hsp in alignment.hsps:

list1 = [hsp.sbjct_start, hsp.frame, hsp.query_start, hsp.sbjct_end]

hsp_list.append(list1)

sorted_hsp_list = sorted(hsp_list, key=itemgetter(0))

hsp_list_size = len(sorted_hsp_list)

for i in range (0, hsp_list_size):

start = sorted_hsp_list[i][0]

end = sorted_hsp_list[i][3]

strand = str(sorted_hsp_list[i][1])

query_start = sorted_hsp_list[i][2]

minus = "-"

position_end_list = []

#the transposon has his left arm in the reverse orientation

if (minus in strand):

print("minus strand")

real_end = start

real_start = real_end - size_of_the_transposon

#sequence_to_search

inverted_motif = motifs.create([nine_bp_inverted_repeat])

putative_transposon = genome_record_dict[subject_id].seq[real_start:real_end].upper()

if nine_bp_inverted_repeat in putative_transposon :

#get the first match of the 9 bp instance to get the smallest transposon !!!

for pos, seq1 in inverted_motif.instances.search(putative_transposon):

position_end_list.append(pos)

position_end = real_end - (size_of_the_transposon - position_end_list[-1])

#=========testing the direct repeats

flanking_repeat1 = str(genome_record_dict[subject_id].seq[position_end-size_of_the_direct_repeat:position_end])

flanking_repeat2 = str(genome_record_dict[subject_id].seq[real_end:real_end+size_of_the_direct_repeat])

flanking_repeat2_big = str(genome_record_dict[subject_id].seq[real_end-2:real_end+size_of_the_direct_repeat+2])

print(flanking_repeat1)

print(flanking_repeat2)

missmatch = hamming(flanking_repeat1, flanking_repeat2)

#IMPORTANT : condition : if the direct repeats are at a hamming distance of 1 (one change) or shifted from no more than 2 bp :

if flanking_repeat1 in flanking_repeat2_big or missmatch < 2 :

transposon_start = position_end-size_of_the_direct_repeat

transposon_end = real_end+size_of_the_direct_repeat

transposon = genome_record_dict[subject_id].seq[transposon_start:transposon_end]

count = count+1

f1.write(">transposon_"+ str(count) + "_" + subject_id + "_" + str(transposon_start) + "_" + str(transposon_end))

f1.write("\n")

f1.write(str(transposon)+ "\n")

#the transposon has his left arm in the forward orientation

else:

real_start = start-query_start

real_end = real_start + size_of_the_transposon

#sequence_to_search

inverted_motif = motifs.create([nine_bp_inverted_repeat_rc])

putative_transposon = genome_record_dict[subject_id].seq[real_start:real_end].upper()

if nine_bp_inverted_repeat_rc in putative_transposon :

#get the first match of the 9 bp instance to get the smallest transposon !!!

for pos, seq1 in inverted_motif.instances.search(putative_transposon):

position_end_list.append(pos)

position_end = position_end_list[0]+ real_start + length_nine_bp_inverted_repeat

#=========testing the direct repeats

flanking_repeat1 = str(genome_record_dict[subject_id].seq[real_start-size_of_the_direct_repeat:real_start])

flanking_repeat2 = str(genome_record_dict[subject_id].seq[position_end:position_end+size_of_the_direct_repeat])

flanking_repeat2_big = str(genome_record_dict[subject_id].seq[position_end-2:position_end+2+size_of_the_direct_repeat])

missmatch = hamming(flanking_repeat1, flanking_repeat2)

#IMPORTANT : condition : if the direct repeats are at a hamming distance of 1 (one change) or shifted from no more than 2 bp :

if flanking_repeat1 in flanking_repeat2_big or missmatch < 2 :

transposon_start = real_start-size_of_the_direct_repeat

transposon_end = position_end+size_of_the_direct_repeat

#sequence

transposon = genome_record_dict[subject_id].seq[transposon_start:transposon_end]

count = count+1

f1.write(">transposon_"+ str(count) + "_" + subject_id + "_" + str(transposon_start) + "_" + str(transposon_end))

f1.write("\n")

f1.write(str(transposon)+ "\n")
