# Supplementary material for: Insertion Specificity of the hATx-6 Transposase of Hydra magnipapillata
Source: Front Mol Biosci. 2021 Dec 20;8:734154. doi: 10.3389/fmolb.2021.734154 (PMC8721813; doi:10.3389/fmolb.2021.734154)
Supplement: Supplementary file 5 [file Table3.DOCX]

**Supplementary Table 2:** List of all oligonucelotides used.

| **Primer name** | **Primer Sequence** |
| --- | --- |
| pMAL-C5X-hatx-6-F | GGGATCGAGGGAAGGATGAACGAAAATTCTGAAAAGG |
| pMAL-C5X -hat-6-R | GCCTTTCGTTTTATTTGATCACAGAGATTCTTTTTTCGTC |
| pSYX20 F1330 | GCAGCGAACTGAATGTCAC |
| pSYX20 R4827 | GTTTGCGCATTCACAGTTCTC |
| clacZ_fwd2 | AGAACTGTGAATGCGCAAACCCTGCAGGCTCGAGTTAT |
| clacZ_rev2 | CGTGACATTCAGTTCGCTGCATGACCATGATTACGGATTCAC |
| hATx-6-RE30-pSYXlac fwd | CGGGCATGAATTACGCCTGCAGGCTCGAGTTATTATTAT |
| hATx-6-RE30-pSYXlac rev | GGATTGCAATCCCTAGTTTGCGCATTCACAGTTCT |
| hATx-6-LE30-pSYXlac fwd | GGATTGCAATCCCTAGCAGCGAACTGAATGTCAC |
| hATx-6-LE30-pSYXlac rev | CGGGCATGAATTACGATGACCATGATTACGGATTCACTG |
| LE20 | 5’-pTTAGGGATTGCAATCCCGGGC  3’- ATCCCTAACGTTAGGGCCCG |
| LE30 | 5’-pTTAGGGATTGCAATCCCGGGCATGAATTACG  3’- ATCCCTAACGTTAGGGCCCGTACTTAATGC |
| RE20 | 5’-pTTAGGGATTGCAATCCCGGAT  3’- ATCCCTAACGTTAGGGCCTA |
| RE30 | 5’-pTTAGGGATTGCAATCCCGGATCCCGAGATCC  3’- ATCCCTAACGTTAGGGCCTAGGGCTCTAGG |
